# Sexual dimorphism and natural variation within and among species in the *Drosophila* retinal mosaic

**DOI:** 10.1186/s12862-014-0240-x

**Published:** 2014-11-26

**Authors:** Maarten Hilbrant, Isabel Almudi, Daniel J Leite, Linta Kuncheria, Nico Posnien, Maria DS Nunes, Alistair P McGregor

**Affiliations:** Department of Biological and Medical Sciences, Oxford Brookes University, Gipsy Lane, Oxford, OX3 0BP UK; Present address: Institute for Developmental Biology, University of Cologne, Zülpicher Str. 47b, 50674 Cologne, Germany; Georg-August-University Göttingen, Johann-Friedrich-Blumenbach Institute for Zoology and Anthropology, Department of Developmental Biology, Ernst-Caspari-House (GZMB), Justus-von-Liebig-Weg 11, 37077 Göttingen, Germany

**Keywords:** Sexual dimorphism, Insect vision, Eye morphology, Evolution, Rhodopsins, *Drosophila*

## Abstract

**Background:**

Insect compound eyes are composed of ommatidia, which contain photoreceptor cells that are sensitive to different wavelengths of light defined by the specific rhodopsin proteins that they express. The fruit fly *Drosophila melanogaster* has several different ommatidium types that can be localised to specific retinal regions, such as the dorsal rim area (DRA), or distributed stochastically in a mosaic across the retina, like the ‘pale’ and ‘yellow’ types. Variation in these ommatidia patterns very likely has important implications for the vision of insects and could underlie behavioural and environmental adaptations. However, despite the detailed understanding of ommatidia specification in *D. melanogaster*, the extent to which the frequency and distribution of the different ommatidium types vary between sexes, strains and species of *Drosophila* is not known.

**Results:**

We investigated the frequency and distribution of ommatidium types based on rhodopsin protein expression, and the expression levels of rhodopsin transcripts in the eyes of both sexes of different strains of *D. melanogaster*, *D. simulans* and *D. mauritiana*. We found that while the number of DRA ommatidia was invariant, Rh3 expressing ommatidia were more frequent in the larger eyes of females compared to the males of all species analysed. The frequency and distribution of ommatidium types also differed between strains and species. The *D. simulans* strain ZOM4 has the highest frequency of Rh3 expressing ommatidia, which is associated with a non-stochastic patch of pale and odd-coupled ommatidia in the dorsal-posterior of their eyes.

**Conclusions:**

Our results show that there is striking variation in the frequency and distribution of ommatidium types between sexes, strains and species of *Drosophila*. This suggests that evolutionary changes in the underlying regulatory mechanisms can alter the distribution of ommatidium types to promote or restrict their expression in specific regions of the eye within and between species, and that this could cause differences in vision among these flies.

**Electronic supplementary material:**

The online version of this article (doi:10.1186/s12862-014-0240-x) contains supplementary material, which is available to authorized users.

## Background

The compound eyes of insects exhibit extensive variation in their size and shape, physiology and biochemical ability to detect different wavelengths of light [1,2]. These differences in vision have allowed insects to adapt to a variety of environments and adopt a wide range of life history strategies [3-6]. Compound eye structure is best understood in the model *Drosophila melanogaster*. Lab strains of *D. melanogaster* generally have between 700 and 800 ommatidia in each eye [7]. However, there is variation in eye size among strains of *D. melanogaster* and among *Drosophila* species due to differences in the size and number of ommatidia [8,9].

**Figure 1:**
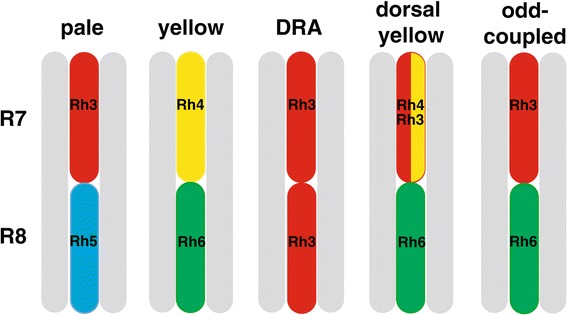
**Schematic representation of five ommatidium types in** ***D. melanogaster*** **.** Grey bars illustrate the rhabdomeres of the six outer photoreceptor cells (R1 to R6) that express the broad range Rh1 and that surround two inner photoreceptor cells (R7 and R8). The ommatidium types are differentiated by distinct combinations of rhodopsin expression in the inner photoreceptor cells, as indicated by coloured bars. Rh3 (red), Rh4 (yellow), Rh5 (blue), Rh6 (green) [13,16,21].

Each ommatidium consists of a cluster of eight light sensitive photoreceptor (PR) cells (R1–R8) and associated cone and pigment cells [7,10]. The six outer PRs (R1 to R6) surround the inner two PRs (R7 and R8) with R7 located closest to the outer surface of the eye, on top of R8 (Figure 1). The PR cells have extensively folded membranes, the rhabdomeres, which contain the rhodopsin proteins [7]. At least five known ommatidium types can be distinguished based on their expression of specific combinations of rhodopsin proteins that are sensitive to different wavelengths of light (Figure 1). All outer PRs in every ommatidium express the broad-range Rhodopsin 1 (Rh1) that enables motion detection, facilitates vision in dim light, and contributes to colour vision [10-12]. The inner PRs enable colour vision and the detection of polarized light [13] (Figure 1). The expression of different rhodopsins in these cells determines the various ommatidium types (Figure 1). In *D. melanogaster,* the ‘yellow’ (y) type makes up 60 to 70% of all ommatidia, and can detect longer wavelengths by expressing the UV-sensitive Rhodopsin 4 (Rh4) in R7 and the green-sensitive Rhodopsin 6 (Rh6) in R8 [14] (Figure 1). The second-most abundant type is the ‘pale’ (p) ommatidia, which account for approximately 30 to 40% of all ommatidia, and express the UV-sensitive Rhodopsin 3 (Rh3) in R7 and the blue-sensitive Rhodopsin 5 (Rh5) in R8, enabling these ommatidia to discriminate among short wavelengths of light [15] (Figure 1). In *D. melanogaster*, the p and y ommatidia are distributed randomly as a consequence of the stochastic expression of underlying regulatory factors [14,16-18].

Two additional types of ommatidia are found in the dorsal region of the eye. At the dorsal rim, a small group of highly specialised dorsal rim area (DRA) ommatidia, express Rh3 in both R7 and R8 to enable sensitivity to linearly polarized UV light [19,20] (Figure 1). More broadly distributed in the dorsal half of the eye, the so-called dorsal-yellow (Dy) ommatidia co-express both Rh3 and Rh4 in R7 (and Rh6 in R8), and are estimated to represent approximately 10% of all ommatidia [21]. A fifth type of ommatidia observed in *D. melanogaster,* which account for approximately 6% of all ommatidia, have been described as ‘odd-coupled’ (OC) because they express Rh3 in R7 (typical for p ommatidia) but Rh6 in R8 (typical for y ommatidia) (Figure 1) [16,22].

Although the specification and regionalisation of ommatidia in the compound eyes of *D. melanogaster* is understood in great detail, relatively little is known beyond this model. There is evidence that the approximate 30 to 70 percent ratio of the p to y ommatidia types is similar to that observed in the house fly *Musca domestica*. However, this apparent conservation based on two species that last shared a common ancestor approximately 100 MYA could be convergent and may belie lineage specific changes and intra-specific variation in this ratio [23-25]. Indeed, we previously reported some evidence for variation in rhodopsin gene expression among species of the *D. melanogaster* complex [9], but any association between the frequency and pattern of ommatidium types, rhodopsin mRNA levels, and eye size remain to be determined.

Here we report our characterisation of the expression and spatial distribution of rhodopsin proteins and the abundance of rhodopsin gene transcripts in the eyes of both sexes of different strains of *D. melanogaster, D. simulans* and *D. mauritiana.* Additionally, we show how this relates to differences in ommatidia number among these species [9]. We found extensive differences between sexes, strains and species in the retinal mosaic of *Drosophila*. These patterns of natural variation can serve as a basis to better understand the development, function and evolution of insect eyes [26].

## Results

### Natural variation in the frequency of ommatidium types

To explore the extent of natural variation in the frequency of ommatidium types among *Drosophila* retinas, we used immunohistochemistry with available antibodies to quantify the number of Rh4-expressing ommatidia compared to Rh3-expressing ommatidia in entire retinas of *D. melanogaster, D. simulans* and *D. mauritiana*. This approach allowed us to distinguish between y ommatidia (including the Dy type), which express Rh4 in R7, p and OC ommatidia, which exclusively express Rh3 in R7, DRA ommatidia, which express Rh3 in both R7 and R8 (Figure 1), and to count the total number of ommatidia.

**Figure 2:**
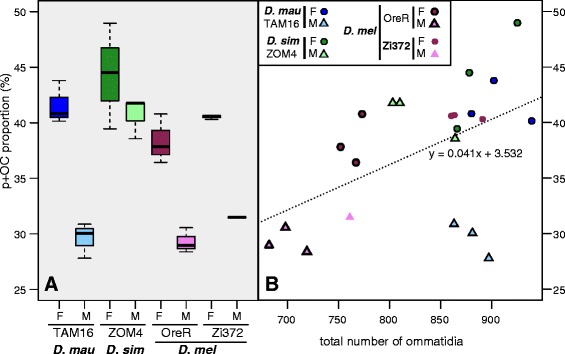
**Variation in the relative proportion of p + OC ommatidia. A**. Boxplots of the percentage of p and OC ommatidia combined, relative to the total of non-DRA ommatidia (y and Dy), specified for strain and species. **B**. Scatterplot of the same statistic as in **A**, derived from the same 22 retinas, as a function of the total number of ommatidia, specified for strain and species. *D. mau*, *D. mauritiana*; *D. sim*, *D. simulans*; *D. mel*, *D. melanogaster*; F, female; M, male.

As expected, we found that the retinas of males of the *D. melanogaster* lab strain Oregon-R were composed of approximately 70% y ommatidia and 30% p + OC ommatidia (Figure 2A; Additional file 1: Table S1). Surprisingly, however, the retinas of *D. melanogaster* Oregon-R females had fewer y ommatidia (61.7% ±2.2) and more p + OC ommatidia compared to males (38.3% ±2.2) (Figure 2A; Additional file 1: Table S1). To test whether the ommatidia type frequencies observed in *D. melanogaster* Oregon-R are representative of other *D. melanogaster* strains, we then also scored the number of Rh4 and Rh3-expressing ommatidia in *D. melanogaster* Zi372, which is an isofemale line that was recently collected from the ancestral range of this species in Africa. We found that *D. melanogaster* Zi372 had a similar, albeit slightly higher proportion of p + OC ommatidia compared to *D. melanogaster* Oregon-R, and furthermore, that this strain was also sexually dimorphic with females having a higher proportion of p + OC ommatidia than a male specimen (40.5% ±0.2 versus 31.5%) (Figure 2A; Additional file 1: Table S1).

We then characterised the composition of the retinal mosaic in *D. simulans* ZOM4 and *D. mauritiana* TAM16, since these species have been previously shown to differ in eye size, morphology and ommatidium number from *D. melanogaster* [9]. Interestingly, these two species also exhibited a sexual dimorphism consistent with that observed with *D. melanogaster* (Figure 2A; Additional file 1: Table S1). Moreover, the proportion of p + OC ommatidia observed for *D. simulans* ZOM4 females (44.3% ±4.8) and males (40.7% ±1.8) was higher than for any other strain that we studied (Figure 2A; Additional file 1: Table S1).

To test for the effects of sex and strain on the proportion of p + OC ommatidia, we constructed a linear fixed effects model that allowed for both additive and interactive effects of these two factors on the observed proportions of ommatidium types among all the retinas that we characterised. This model showed that, overall, males have a significantly lower proportion of p + OC ommatidia than females (F_(1,14)_, F = 66.16, p = 1.13e^−06^). In addition, we found that the factor strain also had a significant effect in this model (F_(3,14)_ = 15.44, p = 0.0001) mainly as a consequence of including *D. simulans* ZOM4. However, despite the high male *D. simulans* ZOM4 values, the interaction between strain and sex was not significant (F_(3,14)_ = 3.312, p =0.05), indicating that this effect is consistent across all strains.

### Variation in total ommatidium number between sexes, strains and species

Consistent with our previous findings [8,9], in this dataset, the total number of ommatidia per retina also differed significantly between strains (linear model, F_(4,17)_ = 38.61, p = 2.55e^−08^) (Additional file 1: Table S1; Additional file 2: Figure S1). For females, *D. mauritiana* TAM16 and *D. melanogaster* Oregon-R had the highest (923.8 ± 29.8) and lowest (761.9 ± 10.8) number of ommatidia respectively. Males had significantly fewer ommatidia than females (−60.2 ± 11.5(SE), F_(1,17)_ = 27.56, p =6.52e^−05^), and this dimorphism was most pronounced in *D. melanogaster* Zi372, and least pronounced in *D. mauritiana* TAM16 (Additional file 1: Table S1; Additional file 2: Figure S1).

It follows from these results that the proportion of p + OC ommatidia and the total number of ommatidia appear to be positively correlated (Figure 2B) (R^2^ = 0.26, F_(1,20)_ =7.11, p =0.01). However, there are clearly outliers suggesting that the overall correlation we have found between p + OC and total number of ommatidia may mask sex and strain specific effects. For example, *D. mauritiana* TAM16 males do not appear to conform to this pattern.

Surprisingly, in contrast to the variation observed in the proportion of p + OC ommatidia, the number of DRA ommatidia varied very little (40.0 ± 3.4) among all of the retinas examined and was not correlated with species, strain or sex (linear model, F_(4,17)_ =0.21, p =0.931) or the total number of ommatidia (Additional file 1: Table S1).

### Spatial distribution of ommatidium types over the retina

**Figure 3:**
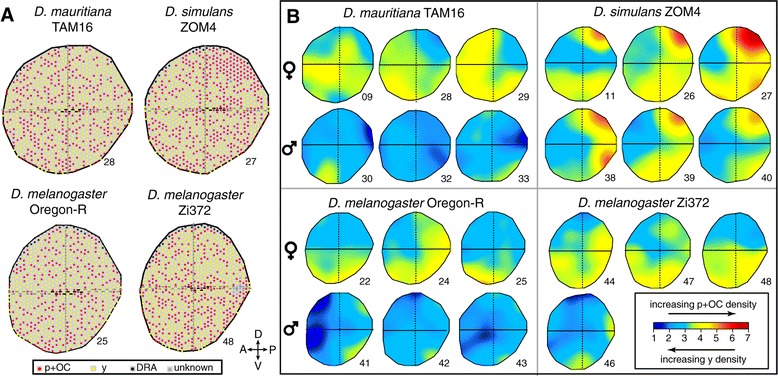
**Variation in the density of p + OC ommatidia over the retina. A**. Representative retinal mosaic maps of one female eye for each of four surveyed strains. Red dots indicate p + OC ommatidia, yellow dots y ommatidia, black dots DRA and grey dots ommatidia of unknown type. Black horizontal stripes indicate the observed equator in the center of the retina, grey horizontal strips the equator extrapolated towards the periphery. Vertical grey dashed lines indicate the center-most dorsal ventral row. **B**. Kernel smoothed density estimates of p + OC ommatidia, and conversely of y ommatidia, in retinal mosaic maps representing both sexes of three *Drosophila* species. Scale bar density values are arbitrary (see Methods) but linear, indicating a seven-fold difference in p + OC density between either end of the scale. Numbers in both **A** and **B** refer to the ID of individual retinas. Retinas 25, 27, 28 and 48 are depicted in both **A** and **B**.

Retinal mosaic maps (Figure 3A; Additional file 3: Figure S2; Additional file 4: Figure S3; Additional file 5: Figure S4; Additional file 6: Figure S5, and see methods) showed that, as expected, all DRA ommatidia are located at the dorsal rim of all retinas, with the odd exception where a few DRA ommatidia were found in the ventral eye (e.g. retina ID: 25, Figure 3A). However, although p + OC and y ommatidia are thought to be distributed stochastically [14], we observed local clustering of p + OC and y ommatidium types in distinct areas of some retinas. In particular, *D. simulans* ZOM4 showed a high incidence or patch of Rh3 expressing ommatidia in the dorsal-posterior region of the retinas of both sexes (Figure 3A; Additional file 4: Figure S3).

To visualise patterns of ommatidium type distribution, we plotted for all retinal mosaic maps the kernel smoothed estimate of the local density of p + OC ommatidia (and hence conversely of y ommatidia) (Figure 3B). This procedure not only highlighted the accentuated cluster of Rh3 expressing ommatidia in the dorsal-posterior of *D. simulans* ZOM4 retinas, but also revealed more subtle variation in spatial density among other retinas. For example, most of the retinas studied exhibited a slight elevation of p + OC density near the ventral rim of the retina (Figure 3B). This is apparent in both female and male retinas, despite the lower overall proportion of p + OC ommatidia in males. A second pattern that emerged from this analysis was an increase in the density of the y type in the anterior retinas of males in particular (Figure 3B). In contrast, *D. mauritiana* TAM16 and *D. melanogaster* Zi372 females appeared to exhibit a deficit of y ommatidia in the anterior (Figure 3B).

We then tested for global (i.e. across the whole retina) clustering of p + OC and/or y ommatidia, by first tallying the number of directly juxtaposed [p + OC]-[p + OC], y-y and [p + OC]-y ommatidia (Additional file 7: Figure S6) and then comparing these numbers with those predicted under the null hypothesis of no spatial dependence between ommatidium types (Additional file 8: Table S2). This approach confirmed that all of the *D. simulans* ZOM4 retinas showed spatial autocorrelation, with significantly reduced numbers of juxtaposed [p + OC]-y ommatidia (retina ID: 11, 26, 27, 38, 39, 40), in combination with an increase in adjoining y-y ommatidia (retina ID: 11, 26, 27, 38, 40) and/or an increase in adjoining [p + OC]-[p + OC] ommatidia (retina ID: 11, 27, 38, 40). The retinas from the other species and strains did not show global spatial autocorrelation, with the exception of *D. melanogaster* Oregon-R (retina ID: 24, 41, 43) and Zi372 (retina ID: 48), which exhibited moderate clustering of y ommatidia, but not of p + OC ommatidia.

**Figure 4:**
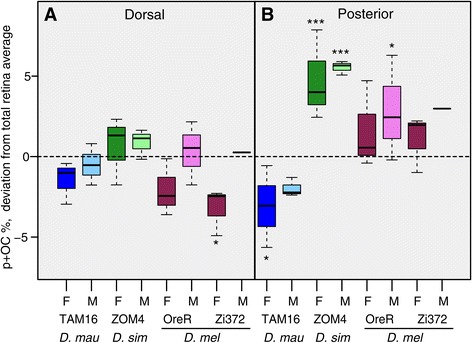
**Variation in the proportion of p + OC ommatidia in the dorsal and anterior retina.** Boxplots showing, per strain and per sex, the percentage of p and OC combined, relative to the total of non-DRA ommatidia in **A** the dorsal retina and **B** the anterior retina. Values in both panels are expressed as the deviation from the percentage of the total retina. Hence, the inverse of the values in **A** give the deviation in the ventral retina, and the inverse of the values in **B** the deviation in the posterior retina. Asterisks indicate level of significance (see main text).

Next we tested for dorsal-ventral patterns in the enrichment of p + OC ommatidia, after partitioning each retina using the equator (Figure 4A; Additional file 3: Figure S2; Additional file 4: Figure S3; Additional file 5: Figure S4; Additional file 6: Figure S5). The percentage of p + OC ommatidia in the dorsal did not deviate significantly from the average across the whole retina, with the exception of the *D. melanogaster* Zi372 female retinas, in which the ventral region was more enriched in p + OC ommatidia than the dorsal region (Figure 3B; Figure 4A; Additional file 9: Table S3). In contrast, comparisons between the anterior and posterior retina halves, using the centre of the dorsal-ventral midline (Figure 3A; Additional file 3: Figure S2; Additional file 4: Figure S3; Additional file 5: Figure S4; Additional file 6: Figure S5), showed more pronounced differences (Figure 4B). Among male and female *D. simulans* ZOM4 retinas the posterior half contained significantly more p + OC ommatidia than the anterior half (Additional file 9: Table S3). *D. melanogaster* Oregon-R males showed the same trend but the difference between anterior and posterior halves was not significant. Interestingly, female *D. mauritiana* TAM16 retinas also showed a significantly skewed anterior-posterior distribution, but with a pattern in the opposite direction from that observed in the other strains (Figure 4B; Additional file 9: Table S3).

To further characterise the dorsal-posterior patch of Rh3 expressing ommatidia observed in *D. simulans* ZOM4, we carried out an additional immunohistochemistry assay in this strain using antibodies against Rh3 and Rh6 to distinguish between p and OC ommatidia. This experiment revealed that this dorsal-posterior patch found in *D. simulans* ZOM4 is actually a mosaic of p and OC ommatidia (Additional file 10: Figure S7).

### Variation in rhodopsin mRNA levels

Our characterisation of ommatidium types based on assaying their expression of Rh3 and/or Rh4 using immunohistochemistry, as described above, suggests that there is extensive natural variation in the retinal mosaics of *D. melanogaster, D. simulans* and *D. mauritiana*. However, wider surveying of natural variation in the expression of rhodopsins requires higher throughput strategies. Quantitative real-time PCR (qPCR) is an approach that has been commonly applied to measure the relative expression of opsins in vertebrates [27-29], and although this approach does not provide any insight into the spatial distribution of PR cells with differential rhodopsin expression, this technique could be applied to further survey natural variation in the expression of rhodopsin genes in *Drosophila*. Therefore, we expanded on our previous experiments [9] and sought to determine if the differential rhodopsin protein expression we have used to score variation in the frequencies of ommatidium types is reflected in relative transcript abundance among sexes, strains and species of *Drosophila* using qPCR (Additional file 11: Table S4).

**Figure 5:**
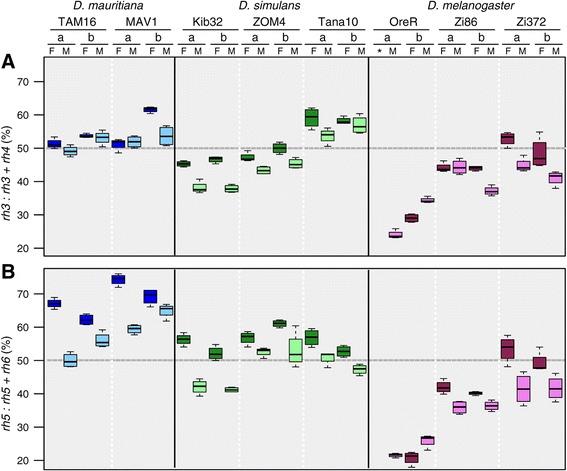
**Sexual dimorphism and variation between** ***Drosophila*** **species and strains in rhodopsin mRNA expression. A** Variation in the expression of *rh3* mRNA relative to the expression of *rh3* and *rh4* combined (*rh3:rh3 + 4*), the *rh3* index. **B** Variation in the expression of *rh5* mRNA relative to the expression of *rh5* and *rh6* combined (*rh5:rh5 + 6*), the *rh5* index. Boxplots represent the variation between four technical replicates within each of biological replicates a and b for each strain. Asterisk, missing data; F, female; M, male; dark blue, female *D. mauritiana*; light blue, male *D. mauritiana*; dark green, female *D. simulans*; light green, male *D. simulans*; dark violet, female *D. melanogaster*; violet, male *D. melanogaster*.

To circumvent the challenge of defining housekeeping genes that would reliably scale with the size of the adult retina across sexes and species, we measured the relative expression of *rh3* mRNA to the expression of *rh3* and *rh4* combined (*rh3:rh3 + 4,* the *rh3* index, Figure 5A), and the relative expression of *rh5* mRNA to the expression of *rh5* and *rh6* combined (*rh5:rh5 + 6,* the *rh5* index, Figure 5B). Since expression of *rh3* and *rh4*, and *rh5* and *rh6* distinguishes the R7 and R8 cells of p + OC and y ommatidia respectively, these expression indexes were thus used as proxies to estimate relative frequencies of ommatidium types in the same strains and species of the *D. melanogaster* complex used above, as well as in several additional strains.

For *D. melanogaster* Oregon-R, we found values of the *rh3* index of 29.2% ±5.6 and 29.0% ±1.1 for males and females respectively. The values of the *rh5* index for *D. melanogaster* Oregon-R males and females were slightly lower at 23.8% ±2.7 and 20.7% ±2.0. However, these qPCR values were broadly consistent with the frequencies of p + OC types we found for *D. melanogaster* Oregon-R (males: 29.3% ±1.13; females: 38.3% ±2.2; Figure 2A; Additional file 1: Table S1).

We then investigated rhodopsin transcript abundance in *D. melanogaster* Zi372 and an additional strain Zi86. These two strains had significantly higher values for the *rh3* index (Zi86, *Χ*^2^ **=** 74.9, p <2e-16; Zi372, *Χ*^2^ **=** 95.7, p <2.2e-16) and the *rh5* index (Zi86, *Χ*^2^ **=** 201.3, p <2e-16; Zi372, *Χ*^2^ **=** 161.8 p <2.2e-16) than *D. melanogaster* Oregon-R (Figure 5). Although this is consistent with the greater proportion of p + OC ommatidia in *D. melanogaster* Zi372 than Oregon-R based on immunohistochemistry (Figure 2A, Additional file 1: Table S1), the relative abundance of both *rh3* (females: 50.6% ±4.1; males: 42.9% ±2.8) and *rh5* (females: 51.3% ±4.0; males: 41.6% ±3.9) transcripts appears to be elevated in the former strain with respect to the number of p + OC ommatidia (females: 40.5% ±0.2; male: 31.5%) (Figure 2A; Figure 5; Additional file 1: Table S1).

We next investigated relative transcript abundance among three strains of *D. simulans* (ZOM4, Tana10 and Kib32) and two *D. mauritiana* strains (TAM16 and MAV1) (Figure 5). We found that the three *D. simulans* strains were all significantly different from each other for both the *rh3* index (*Χ*^2^ **=** 432.9, p <2.2e-16) and the *rh5* index (*Χ*^2^ **=** 51.8, p =5.6e-12) (Figure 5). In addition, *D. mauritiana* MAV1 had significantly higher *rh3* (*Χ*^2^ **=** 9.7, p =1.9e-3) and *rh5* (*Χ*^2^ **=** 45.8, p =1.29e-11) indexes than *D. mauritiana* TAM16 (Figure 5).

For *D. simulans* ZOM4 the *rh3* indexes (females, 48.9% ±2.0; males, 44.4% ±1.6) were consistent with the frequency of p + OC ommatidia (females, 44.3% ±4.8; males, 40.7% ±1.8), but the *rh5* indexes (females, 58.9% ±2.7; males, 52.8% ±3.6) were somewhat elevated in comparison. For *D. mauritiana* TAM16, the values of both the *rh3* (females, 52.5% ±1.6; males, 51.1% ±2.7) and *rh5* indexes (females, 64.7% ±3.0; males, 53.1% ±3.82) were also elevated compared to the frequency of p + OC ommatidia (females, 41.6% ±1.9; males, 29.6.% ±1.6), particularly for *rh5* and especially in the case of males. This analysis shows that the qPCR data generally gives a similar trend to the ommatidium type frequencies measured using immunohistochemistry, especially for *D. melanogaster;* for the other species the transcript abundance of *rh5* in particular appears to be elevated.

The trend in the qPCR data for most strains suggested that males and females differed for the values of *rh3* and *rh5* indexes consistent with dimorphism in p + OC ommatidia observed from the antibody stainings against rhodopsin proteins. To test this further we constructed a linear mixed model to compare genders and at the same time determine whether the qPCR data showed any differences between species, while treating the factor strain as a random effect (i.e. as replicates of the factor “species”). This showed that both the *rh3* and *rh5* indexes differed significantly between sexes, with females having higher values than males in both cases (*X*^2^_(1)_ = 41.35, p = 1.27e-10, and *X*^2^_(1)_ = 91.21, p <2.2e-16, respectively) (Additional file 12: Table S5; Additional file 13: Table S6). While the *rh3* index values were not significantly different in this linear mixed model for species (*X*^2^_(2)_ = 3.14, p = 0.208), *rh5* index values did vary significantly between species (*X*^2^_(2)_ = 10.60, p = 0.005) (Additional file 12: Table S5; Additional file 13: Table S6) with the *D. mauritiana* and *D. melanogaster* strains exhibiting the highest and lowest levels of p ommatidium-type associated rhodopsin mRNA expression respectively.

Finally, to more directly compare the qPCR results of the current study with those obtained previously [9], we calculated the expression of *rh3*, *rh4* and *rh6* relative to the total of these three rhodopsin mRNA (omitting the *rh5* expression data). In this representation, and consistent with previous results, *D. melanogaster* Oregon-R shows the smallest relative proportion of *rh3* mRNA, whereas both *D. mauritiana* strains (TAM16 and MAV1) exhibit high relative proportions (Additional file 14: Figure S8). Moreover, the relative proportion of *rh3* expression in *D. simulans* Kib32 is intermediate between *D. mauritiana* and *D. melanogaster* Oregon-R (Additional file 15: Figure S8). However, compared to our previous study, our current data also highlights the variation between strains - both within *D. simulans* and *D. melanogaster* (Additional file 14: Figure S8).

## Discussion

The *Drosophila* compound eye consists of a mosaic of different ommatidium types. Each type expresses a different combination of rhodopsins (Figure 1), and is therefore sensitive to different fractions of the light spectrum. Accordingly, the relative proportions and the spatial arrangement of these different types are important aspects of the vision of these flies [12,15]. The regulation and development of retinal regionalisation are well understood in *D. melanogaster*. In this species, specialised DRA ommatidia are positioned dorsal to a mosaic of stochastically distributed p and y ommatidia. Our results show, however, that there is extensive natural variation in ommatidium type frequencies and distributions, as well as in the expression of rhodopsin transcript levels among sexes, strains and species of the *D. melanogaster* species subgroup.

### Sexual dimorphism in rhodopsin expression

Both our antibody staining and qPCR assays to quantify ommatidia type frequencies and distributions showed that there are differences in the expression of rhodopsins between the sexes of all three *Drosophila* species. Specifically, females express more p type associated rhodopsin mRNA (*rh3* and *rh5*) than males (Figure 5), which is reflected in the higher proportion of p + OC ommatidium types in females compared to males (Figure 2A). This could mean that overall the vision of male *Drosophila* is different from that of females.

Sexual dimorphism in photoreceptor fate has been described in *Musca*, for example the evolution of Rh1 expression in the inner photoreceptors of a subset of ommatidia in males [25], which is associated with higher spatial and temporal resolution, allowing males to detect small moving objects better than females [10,30]. However, the potential functional consequences of the more subtle variation in the retinal mosaic that we have found in the case of *Drosophila* requires further testing. An alternative but not mutually exclusive explanation is that the dimorphism observed in *Drosophila* is a consequence of differences in ommatidium number seen between males and females, which cannot be entirely accounted for by the difference in body size between sexes [9]. This explanation is supported by our finding of a positive relationship between the proportion of p + OC ommatidia and total ommatidium number across species and sexes (Figure 2B). However, the regulatory mechanisms underlying this relationship, remain to be found and may differ between lineages since male *D. mauritiana* TAM16 retinas do not fit the above hypothesis because they exhibit high numbers of ommatidia but a relatively low proportion of them are p + OC.

Interestingly, the number of DRA ommatidia varied very little, did not correlate with gender, and did not increase even in retinas with larger numbers of ommatidia in total. This suggests that there are constraints on the number of these ommatidia, possibly as a consequence of the limited range of expression of the underlying regulatory factors, such as *homothorax,* when this ommatidium type is defined during pupation [31].

### Natural variation in rhodopsin expression within and among species

Current knowledge of the molecular mechanisms underlying ommatidium type specification is centred on findings that the p and y ommatidia fate is determined stochastically [17,22]. The strongest support for this hypothesis was found by characterising the arrangement of p and y ommatidia in 28 different *D. melanogaster* retinas [14]. However, since in this previous study only a sample of ommatidia were investigated per retina (about 150), it is not entirely clear what region of each retina was surveyed, and if males, females or both sexes were used. Our data therefore builds on that of Bell et al. [14] by characterising the mosaic map of the whole retina of each specimen, for males and females separately. Spatial analysis of complete retinal mosaic maps shows that p + OC and y ommatidia are not just uniformly spread over the eye, but that there are localized accumulations as well as regional variations in the density of these ommatidium types (Figures 3 and 4).

One of our most striking observations is the high overall percentage of p + OC ommatidia in *D. simulans* ZOM4 (Figure 2A), which correlates with the occurrence of a patch of p + OC ommatidia types in the dorsal-posterior retina of both sexes of this strain (Figure 3), and a generally high density of Rh3 expressing ommatidia in the posterior retina (Figure 4B). However, to test if this patch is strain specific or commonly found in *D. simulans* requires further systematic study of multiple retinas of both sexes of a range of *D. simulans* strains.

Thus, although our data does not refute that p and y specification is stochastic across large parts of the retina, it does provide further evidence that the stochastic p to y switch could be modulated regionally, as shown previously [18]. Indeed, there might be other regional influences from as yet unknown factors that influence this switch and allow for the enrichment and conversely deficiency of types in different parts of the eye such as the enrichment and paucity of p + OC ommatidia in the posterior of the retinas of *D. simulans* ZOM4 and *D. mauritiana* TAM16 respectively (Figure 3; Figure 4B). One intriguing possibility is that these variations are due to differences in the expression of *spineless*, which represses p fate and promotes y fate [17,22].

We found variation in ommatidium frequency and distribution among *Drosophila* strains and species based on immunohistochemistry (Figure 2A), supported to some extent by the trends in the relative levels of rhodopsin mRNA expression (Figure 5; Additional file 14: Figure S8). These results, which corroborate our previous findings [9], suggest that there might be differences in vision among these flies. However, the visual function, behavioural consequences or even adaptive reasons for this variation remain to be tested. Natural variation in opsin mRNA expression has been the focus of numerous studies in cichlid fishes, and has been shown to correlate with behavioural and environmental factors such as foraging and ambient light [28]. On the other hand, variation in stochastic cone ratios has also been described between human individuals, but does not seem to correlate with variation in colour vision [32,33]. A range of assays for studying the effects of genetic manipulation on *Drosophila* visual behaviour have been developed [15,34], and could provide a starting point for behavioural studies of natural variants. The results could be particularly insightful since although the ecology of these species is rather enigmatic, at least *D. melanogaster* and *D. simulans* are world-wide distributed commensal generalists and as such have adapted to a wide range of habitats since their evolution in Africa and Madagascar [35-40].

Despite the promise of high-throughput data acquisition via qPCR and successful application of this technique in other organisms, our current survey of variation in rhodopsin mRNA expression in *Drosophila* highlights some issues with this approach. First, the relationship between mRNA and protein expression levels is generally unclear and difficult to predict [41,42], and would need to be studied for the particular relationship between *Drosophila* rhodopsin transcripts and proteins before accurate inferences could be made between the two. Second, even if rhodopsin protein levels could be estimated efficiently and accurately via qPCR, this does not directly translate to the relative proportions of ommatidium types that we observed because, for example, p ommatidia might not express as much Rh3 as y ommatidia express Rh4, and DRA, OC and Dy types are likely to have a complex influence on relative rhodopsin levels.

Given these challenges, it is remarkable and encouraging that our qPCR and antibody based surveys identified similar trends, showing both a significant sexual dimorphism and significant differences between strains. Hence, for future studies, the two approaches could provide potentially complementary ways to study variation in vision. For example our qPCR assay could be further developed to quickly and reliably determine transcript levels in single flies, which would open up the possibility to test the genetic differences underlying variation in rhodopsin mRNA expression.

## Conclusions

We present here, to our knowledge, the most comprehensive survey to date of rhodopsin variation among sexes, strains and species of the *D. melanogaster* species subgroup. Our results suggest that natural variation within and among species in as yet unknown regulatory mechanisms can alter the local distribution of ommatidium types described in the model *D. melanogaster*. Future studies of the genetic basis of the differences in ommatidia frequencies and distributions that we have found will allow further elucidation of differences in gene regulation between sexes and evolutionary differences in cell fate within and between species.

## Methods

### Drosophila strains and culture

We employed two *D. mauritiana* strains (TAM16, MAV1 collected in Mauritius in 2007 and 2009, respectively [43])*,* three *D. simulans* strains (ZOM4 and Kib32 collected in 2001 in Malawi and Uganda, respectively [44] and Tana10 collected in Madagascar 2008 and kindly provided by J. David) and three *D. melanogaster* strains (Oregon-R, and Zi86 and Zi372 collected in Zambia in 2010 and kindly provided by J. Pool). All flies were raised on a standard cornmeal diet at 25**°**C.

### Retinal mosaic maps: construction and analysis

We dissected 22 adult retinas from males and females of *D. mauritiana* TAM16, *D. simulans* ZOM4, and *D. melanogaster* Oregon-R and Zi372. Retinas were stained with antibodies against Rh3 and Rh4, in order to distinguish between DRA, p + OC and y ommatidia, and with phalloidin, to visualize rhabdomeres. Subsequently, a retinal mosaic, composed of these three ommatidium types, was manually mapped for each of the retinas, and the number of each type of ommatidia was then deduced from these retinal mosaic maps. *D. simulans* ZOM4 retinas were also stained with antibodies against Rh3 and Rh6 respectively.

Adult flies for dissection were collected 9–13 days after eclosion from non-crowded standard culture bottles kept at a 12 hours light cycle. Flies were sedated with CO_2_ and kept on ice until dissection. One retina per fly was prepared for antibody staining by removing all internal tissue from the head capsule, including the optic lobes and the medulla, as well as surrounding cuticle, as described previously [45]. Retinas were blocked for one hour with PBS + 0.3% Triton-X-100 (PBT) supplemented with 5% normal goat serum. Next, they were incubated overnight at room temperature (RT) with a mixture of primary antibodies: mouse-anti-Rh3 [14: IgG1, clone 2B1] (1:20) and rabbit-anti-Rh4 (from C. Zuker/ N. Colley) (1:40) or rabbit-anti-Rh3 (from C. Zuker/ N. Colley) (1:10) and mouse-anti-Rh6 (clone 9D12, IgG1) (from Steven Britt) (1:40) in PBT. After 4 × 2 hrs PBT wash steps, retinas were incubated overnight with a mixture of secondary antibodies: alexa 647 conjugated anti-mouse (Invitrogen) (1:200), cy3 conjugated anti-rabbit (Invitrogen) (1:200) and alexa 488 conjugated phalloidin (Invitrogen) (1:50) at RT. After 2 × 1 hr PBT wash steps retinas were mounted in Prolong Gold (Invitrogen). Mounting medium was left to solidify for at least 16 hrs before imaging.

**Figure 6:**
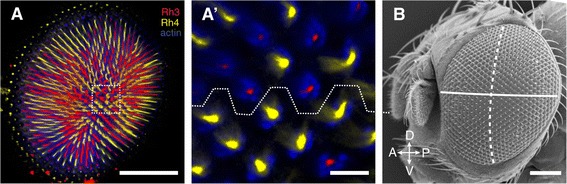
**Construction of retinal mosaic maps. A**. Single slice of a confocal laser scanning stack (acquired with a 25× objective), showing about half of all ommatidia, in the centre of a male *D. simulans* ZOM4 retina. p + OC ommatidia are distinguished by staining with anti-Rh3 (red) from y ommatidia stained with anti-Rh4 (yellow). Actin (blue) staining visualizes the rhabdomeres. **A'**. The equator (white dashed line) in the boxed region in A at higher magnification (40× objective). **B**. SEM micrograph of a ZOM4 male head with the approximate location of equator (solid line) and dorsal-ventral midline (dashed line) indicated. Anterior is to the left. Scale bars A and C: 100 μm, A': 10 μm.

For retinal mosaic map reconstruction, confocal Laser Scanning Microscopy (CLSM) was performed on a Zeiss LSM 510 META using a Zeiss Plan-Neofluar 25×/0.80 objective (Figure 6A). Pinhole size, gain and intensities of excitation lasers at 488, 543 and 633 nm were adjusted per specimen and per region of the retina in order to obtain the best signal-to-noise ratio. Due to the size of the retinas, multiple stacks were obtained per retina and, depending on the complexity of the region, the z-stack interval was set to 2–4 μm. Stacks were converted from LSM-files to TIF-files (RGB) in Fiji [46]. The equator was identified in the centre of each retina via phalloidin (actin) staining of ommatidium rhabdomeres using a Zeiss EC Plan Neofluar 40×/1.30 objective (Figure 6A’). Subsequently, retinal mosaics were interpreted manually from the CLSM scans and recorded on a hexagonal grid. Distinction was made between DRA (Rh3 expression in both R7 and R8 cells), p + OC (Rh3 expression in R7 cells only) and y (Rh4 expression in R7 cells only) ommatidia. Note that, since the Rh4 signal was generally stronger than the Rh3 signal, co-expression of Rh3 and Rh4 in R7 cells (indicative of Dy ommatidia, Figure 1), could not always be distinguished unambiguously, and hence these ommatidia were conservatively scored as y. A small percentage (2.3% in one case, but typically lower, see Additional file 1: Table S1) of all ommatidia could not be assigned to any of these types, and was scored as “unknown”. Finally, the position of the equator was extrapolated anteriorly and posteriorly, and the centre-most dorsal-ventral row was marked (Figure 3A; Additional file 3: Figure S2; Additional file 4: Figure S3; Additional file 5: Figure S4; Additional file 6: Figure S5) to define anterior and posterior. Scanning electron microscopy (SEM) shows how this equator-based coordinate system relates to landmarks on the head capsule (Figure 6B) (for SEM methods see [9]).

y, p + OC, DRA and unknown ommatidia were counted from the retinal mosaic maps and the percentage of p + OC ommatidia relative to p + OC and y combined was calculated (Additional file 1: Table S1). We then fitted a linear model in R, on both this p + OC percentage and the total number of ommatidia per retina, allowing for additive and interactive effects of strain and sex.

For spatial data analysis, the dimensions of all retinal mosaic maps were first standardised by precisely overlaying all hexagonal grids and cropping to 1400×1400 pixels. Next, the centres of the scored ommatidia were converted to x,y coordinates using the centroid option of Fiji’s “Analyze particles” function. With these, the R package **spatstat** 1.36-0 [47] was used to create a marked spatial point pattern and a convex hull shape for each retina (Figure 3A; Additional file 3: Figure S2; Additional file 4: Figure S3; Additional file 5: Figure S4; Additional file 6: Figure S5), and to compute and plot kernel smoothed density estimates of p + OC ommatidia using the *density.ppp* function (Figure 3B). In order to generate comparable plots for all retinas, the way numerical density values were mapped to colours was fixed using the *zlim* option (ranging from 0.0001 to 0.0007 points per pixel). However, retinal mosaics are not truly point processes, since the different ommatidium types are registered onto a (hexagonal) grid. Therefore, in order to further investigate the distribution of p + OC and y ommatidia, Dirichlet tessellations of the marked point patterns were converted to spatial polygons objects as implemented in the package **sp** 1.0-14 [48] and subsequently analysed with **spdep** 0.5-71 [49]. First, DRA ommatidia were removed from each retina since it is well established that these are clustered at the dorsal rim of the eye [50-52]. Next, ommatidium neighbour relations were defined based on contiguity, with binary weights (i.e. each ommatidium has six neighbouring ommatidia - edges excluded- and interactions between adjoining ommatidia are equally strong). We then used the *joincount.multi* function, which tallies join counts between same-type (yellow-yellow, pale-pale) and different-type (yellow-pale) ommatidia, to test for global spatial autocorrelation (Additional file 7: Figure S6).

Finally, we divided each retinal mosaic into dorsal and ventral halves (using the equator), scored ommatidium subtypes in each of these compartments and tested for deviation from homogeneity of the ratio of p + OC:Y using repeated G-tests of goodness-of-fit, after pooling counts for individual retinas for each sex and strain (since tests for heterogeneity were not significant). This procedure was repeated after dividing retinas in anterior and posterior halves using the centre-most dorsal-ventral row.

### qPCR: sample collection and analysis

To control for potential age and circadian effects, two biological replicates (A and B) were collected per strain: at 14 and 15 days after eclosion, between 1–3 pm. Six culture vials were raised per biological replicate. Larval density was controlled by limiting the content of each vial to 40 freshly hatched L1 larvae. Animals were raised in darkness. Vials from each replicate were pooled on collection, and males and females were directly flash frozen in separate vials using liquid nitrogen. Subsequent vortexing and sieving allowed for the separation of heads from remaining body parts, as well as for stripping antennae and bristles from the heads [9].

Total RNA was extracted from heads using RNeasy mini (QIAGEN; animal tissue protocol; disruption by squashing heads with a disposable pestle in buffer RLT). Yield was quantified using a Nanodrop 1000 spectrophotometer (Thermo Scientific), and 0.7-0.9 μg of total RNA was added to a 40 μl *DNAse*I digestion reaction (Thermo Scientific). Reverse transcription was performed with a RevertAid First Strand cDNA synthesis kit (Thermo Scientific) using oligo(dT)_18_ primers in a double (40 μl) standard reaction including 22 μl DNaseI treated total RNA. In parallel, reactions without the RT enzyme were set up. cDNA was diluted 1:5 with ddH_2_O and half of the volume was diluted further to 1:15. 15 μl PCR reactions were set up in 96 well 4titude FrameStar plates, avoiding outer wells: 7.5 μl Maxima SYBR Green 2× master mix (Thermo Scientific), 5 μl template, 1.7 μl H_2_O, 0.25 μl primers (10 μM) each, and 0.3 μl Uracil-DNA Glycosylase (Thermo Scientific). Published primer sequences were used for quantifying *rh3*, *rh4* and *rh6* [9], new primers were designed for *rh5* (Additional file 15: Table S7). Reactions were performed on a Bio-Rad CFX96 thermo cycler (Additional file 16: Table S8). Primer efficiency of the different pairs (Additional file 15: Table S7) was determined using Bio-Rad CFX Manager 3.0, with 1:4, 1:16, 1:32 and 1:64 dilution series of cDNA (1:5 dilutions). qPCR was performed in quadruplicates using the 1:15 cDNA dilutions of each extraction. Cq values were calculated with Bio-Rad CFX Manager 3.0 in Single Threshold mode.

Analogous to methods described previously [53], two relative expression indexes were calculated to compare Rhodopsin mRNA expression levels across sexes, strains and species. The first, the *rh3* index (*rh3:rh3 + 4)*, represents the percentage of *rh3* expression relative to the expression of *rh3* and *rh4* combined (Additional file 17: Table S9). Similarly the second, *rh5* index (*rh5:rh5 + 6)*, represents the percentage of *rh5* expression relative to the expression of *rh5* and *rh6* combined.

We analysed the variation in both *rh3:rh3 + 4* and *rh5:rh5 + 6* in R [54] by fitting a linear mixed model (LMM) for each, using **lme4** version 1.1-5 [55]. We modelled species and sex as fixed effects, without interaction term. Intercepts for strains and biological replicates were included as random effects. Visual inspection of residual and normal Q-Q plots did not reveal deviations from homoscedasticity or normality.

## Availability of supporting data

All supporting data are included as additional files.

## Additional files

Additional file 1: Table S1 Counts of all ommatidia. Additional file 2: Figure S1 Total ommatidium number per strain and sex. Additional file 3: Figure S2 Overview of *D. mauritiana* TAM16 retinal mosaic maps. Additional file 4: Figure S3 Overview of *D. simulans* ZOM4 retinal mosaics. Additional file 5: Figure S4 Overview of *D. melanogaster* Oregon-R retinal mosaic maps. Additional file 6: Figure S5 Overview of *D. melanogaster* Zi372 retinal mosaic maps. Additional file 7: Figure S6 Detail of a retinal mosaic map. Lines connecting closed circles indicate joins between contiguous ommatidia. Additional file 8: Table S2 Global spatial autocorrelation test statistics. Additional file 9: Table S3 Test statistics for comparison between dorsal/ventral and anterior/posterior retina compartments. Additional file 10: Figure S7 Pale and Odd-Coupled ommatidia in the *D. simulans* ZOM4 dorsal-posterior retina. Combined immunostaining of Rh3 in R7 cells (left, magenta) and Rh6 in R8 cells (centre, green) show that the patch of p + OC ommatidia identified in the dorsal posterior retina of this strain consists of both p and OC ommatidia (right, overlay). Arrowheads: OC ommatidia, arrows: p ommatidia, asterisks: DRA ommatidia. Scale bar: 50 μm. Additional file 11: Table S4 Cq values for qPCR quantification of *rh3*, *rh4*, *rh5* and *rh6* mRNA. Additional file 12: Table S5
*rh3*:*rh3 + 4* qPCR results: statistics of LMM analyses. Additional file 13: Table S6
*rh5*:*rh5 + 6* qPCR results: statistics of LMM analyses. Additional file 14: Figure S8 Sexual dimorphism and variation between *Drosophila* species and strains in the expression of *rh3*, *rh4* and *rh6* mRNA. Histograms of the relative expression of *rh3*, *rh4* and *rh6*, as a percentage of the total expression of these three rhodopsins, provide an alternative representation of the quantitative real-time PCR shown in Figure 5 of the main text. Compare with Figure 4 in Posnien et al. (2012), in which OreR represented *D. melanogaster*, Kib32 *D. simulans* and TAM16 *D. mauritiana*. Additional file 15: Table S7 Rhodopsin qPCR primer combinations. Additional file 16: Table S8 qPCR thermo cycling program. Additional file 17: Table S9 Equations used to calculate relative *rhodopsin* mRNA indexes.

## References

[1] Elzinga RJ (2003). Fundamentals of Entomology.

[2] Snodgrass EE (1935). Principles of Insect Morphology.

[3] Land MF (1997). Visual acuity in insects. Annu Rev Entomol.

[4] Gonzalez-Bellido PT, Wardill TJ, Juusola M (2011). Compound eyes and retinal information processing in miniature dipteran species match their specific ecological demands. Proc Natl Acad Sci U S A.

[5] Kirschfeld K, Wenk P (1976). The dorsal compound eye of simuliid flies: an eye specialized for the detection of small, rapidly moving objects. Z Naturforsch Section C: Biosciences.

[6] Land MF, Eckert H (1985). Maps of the acute zones of fly eyes. J Comp Phys.

[7] Wolff T, Ready DF, Bate M, Martinez Arias A (1993). Pattern Formation in the *Drosophila* Retina. The Development of Drosophila Melanogaster.

[8] Arif S, Hilbrant M, Hopfen C, Almudi I, Nunes MD, Posnien N, Kuncheria L, Tanaka K, Mitteroecker P, Schlötterer C, McGregor AP (2013). Genetic and developmental analysis of differences in eye and face morphology between Drosophila simulans and Drosophila mauritiana. Evol Dev.

[9] Posnien N, Hopfen C, Hilbrant M, Ramos-Womack M, Murat S, Schönauer A, Herbert SL, Nunes MD, Arif S, Breuker CJ, Schlötterer C, Mitteroecker P, McGregor AP: **Evolution of eye morphology and rhodopsin expression in the Drosophila melanogaster species subgroup.** *PLoS ONE* 2012, **7**(5):e37346. 10.1371/journal.pone.0037346 PMC3360684 22662147

[10] Hardie RC, Ottoson D (1985). Functional Organization of the fly Retina. Progress in Sensory Physiology.

[11] Zuker CS, Cowman AF, Rubin GM (1985). Isolation and structure of a rhodopsin gene from D. melanogaster. Cell.

[12] Schnaitmann C, Garbers C, Wachtler T, Tanimoto H (2013). Color discrimination with broadband photoreceptors. Curr Biol.

[13] Wernet MF, Desplan C (2004). Building a retinal mosaic: cell-fate decision in the fly eye. Trends Cell Biol.

[14] Bell ML, Earl JB, Britt SG (2007). Two types of Drosophila R7 photoreceptor cells are arranged randomly: a model for stochastic cell-fate determination. J Comp Neurol.

[15] Yamaguchi S, Desplan C, Heisenberg M (2010). Contribution of photoreceptor subtypes to spectral wavelength preference in Drosophila. Proc Natl Acad Sci U S A.

[16] Chou WH, Huber A, Bentrop J, Schulz S, Schwab K, Chadwell LV, Paulsen R, Britt SG (1999). Patterning of the R7 and R8 photoreceptor cells of Drosophila: evidence for induced and default cell-fate specification. Development.

[17] Johnston RJ, Desplan C (2014). Interchromosomal communication coordinates intrinsically stochastic expression between alleles. Science.

[18] Thanawala SU, Rister J, Goldberg GW, Zuskov A, Olesnicky EC, Flowers JM, Jukam D, Purugganan MD, Gavis ER, Desplan C, Johnston RJ (2013). Regional modulation of a stochastically expressed factor determines photoreceptor subtypes in the Drosophila retina. Dev Cell.

[19] Fortini ME, Rubin GM (1990). Analysis of cis-acting requirements of the Rh3 and Rh4 genes reveals a bipartite organization to rhodopsin promoters in Drosophila melanogaster. Genes Dev.

[20] Fortini ME, Rubin GM (1991). The optic lobe projection pattern of polarization-sensitive photoreceptor cells in Drosophila melanogaster. Cell Tissue Res.

[21] Mazzoni EO, Celik A, Wernet MF, Vasiliauskas D, Johnston RJ, Cook TA, Pichaud F, Desplan C: **Iroquois complex genes induce co-expression of rhodopsins in Drosophila.** *PLoS Biol* 2008, **6**(4):e97. 10.1371/journal.pbio.0060097 PMC2323304 18433293

[22] Wernet MF, Mazzoni EO, Celik A, Duncan DM, Duncan I, Desplan C (2006). Stochastic spineless expression creates the retinal mosaic for colour vision. Nature.

[23] Franceschini N, Kirschfeld K, Minke B (1981). Fluorescence of photoreceptor cells observed in vivo. Science.

[24] Hardie RC (1983). Projection and connectivity of sex-specific photoreceptors in the compound eye of the male housefly (Musca domestica). Cell Tissue Res.

[25] Franceschini N, Hardie RC, Ribi W, Kirschfeld K (1981). Sexual dimorphism in a photoreceptor. Nature.

[26] Nunes MD, Arif S, Schlötterer C, McGregor AP (2013). A perspective on micro-evo-devo: progress and potential. Genetics.

[27] Carleton KL, Kocher TD (2001). Cone opsin genes of african cichlid fishes: tuning spectral sensitivity by differential gene expression. Mol Biol Evol.

[28] Hofmann CM, O’Quin KE, Marshall NJ, Cronin TW, Seehausen O, Carleton KL: **The eyes have it: regulatory and structural changes both underlie cichlid visual pigment diversity.** *PLoS Biol* 2009, **7**(12):e1000266. 10.1371/journal.pbio.1000266 PMC2790343 20027211

[29] Spady TC, Parry JW, Robinson PR, Hunt DM, Bowmaker JK, Carleton KL (2006). Evolution of the cichlid visual palette through ontogenetic subfunctionalization of the opsin gene arrays. Mol Biol Evol.

[30] Cronin TW, Johnsen S, Marshall NJ, Warrant EJ (2014). Visual Ecology.

[31] Wernet MF, Desplan C (2014). Homothorax and Extradenticle alter the transcription factor network in Drosophila ommatidia at the dorsal rim of the retina. Development.

[32] Roorda A, Williams DR (1999). The arrangement of the three cone classes in the living human eye. Nature.

[33] Hofer H, Carroll J, Neitz J, Neitz M, Williams DR (2005). Organization of the human trichromatic cone mosaic. J Neurosci.

[34] Paulk A, Millard SS, van Swinderen B (2013). Vision in Drosophila: seeing the world through a Model’s eyes. Annu Rev Entomol.

[35] Kopp A, Frank A, Fu J (2006). Historical biogeography of Drosophila simulans based on Y-chromosomal sequences. Mol Phylogenet Evol.

[36] Ballard JW (2004). Sequential evolution of a symbiont inferred from the host: Wolbachia and Drosophila simulans. Mol Biol Evol.

[37] Lachaise D, Silvain JF (2004). How two Afrotropical endemics made two cosmopolitan human commensals: the Drosophila melanogaster-D. simulans palaeogeographic riddle. Genetica.

[38] Begun DJ, Aquadro CF (1993). African and North American populations of Drosophila melanogaster are very different at the DNA level. Nature.

[39] Veuille M, Baudry E, Cobb M, Derome N, Gravot E (2004). Historicity and the population genetics of Drosophila melanogaster and D. simulans. Genetica.

[40] Nunes MD, Neumeier H, Schlötterer C (2008). Contrasting patterns of natural variation in global Drosophila melanogaster populations. Mol Ecol.

[41] de Sousa AR, Penalva LO, Marcotte EM, Vogel C (2009). Global signatures of protein and mRNA expression levels. Mol BioSyst.

[42] Grun D, Kirchner M, Thierfelder N, Stoeckius M, Selbach M, Rajewsky N (2014). Conservation of mRNA and protein expression during development of C. elegans. Cell Rep.

[43] Nolte V, Pandey RV, Kofler R, Schlötterer C (2013). Genome-wide patterns of natural variation reveal strong selective sweeps and ongoing genomic conflict in Drosophila mauritiana. Genome Res.

[44] Nolte V, Schlötterer C (2008). African Drosophila melanogaster and D. simulans populations have similar levels of sequence variability, suggesting comparable effective population sizes. Genetics.

[45] Hsiao HY, Johnston RJ, Jukam D, Vasiliauskas D, Desplan C, Rister J: **Dissection and immunohistochemistry of larval, pupal and adult Drosophila retinas.** *J Vis Exp* 2012, **69:**e4347. 10.3791/4347 PMC3523422 23183823

[46] Schindelin J, Arganda-Carreras I, Frise E, Kaynig V, Longair M, Pietzsch T, Preibisch S, Rueden C, Saalfeld S, Schmid B, Tinevez JY, White DJ, Hartenstein V, Eliceiri K, Tomancak P, Cardona A (2012). Fiji: an open-source platform for biological-image analysis. Nat Meth.

[47] Baddeley A, Turner R (2005). Spatstat: an R package for analyzing spatial point patterns. J Stat Soft.

[48] Bivand RS, Pebesma E, Gomez-Rubio V (2013). Applied Spatial Data Analysis With R.

[49] Bivand RS: **spdep: Spatial dependence: weighting schemes, statistics and models.** [http://CRAN.R-project.org/package=spdep]

[50] Wada S (1974). Spezielle randzonale ommatidien der fliegen (Diptera: Brachycera): architektur und verteilung in den komplexauaen. Zeitschrift Fur Morphologie Der Tiere.

[51] Hardie RC: **Functional Organization of the Fly Retina.** In *Progress in Sensory Physiology*, Volume 5. Edited by Autrum H, Ottoson D, Perl E, Schmidt R, Shimazu H, Willis W. Springer Berlin Heidelberg; 1985:1–79.

[52] Wernet MF, Labhart T, Baumann F, Mazzoni EO, Pichaud F, Desplan C (2003). Homothorax switches function of Drosophila photoreceptors from color to polarized light sensors. Cell.

[53] Carleton KL (2011). Quantification of transcript levels with quantitative RT-PCR. Methods Mol Biol.

[54] R Core Team (2013). R: A Language and Environment for Statistical Computing.

[55] Bates D, Maechler M, Bolker B and Walker S: **lme4: Linear mixed-effects models using Eigen and S4.** [http://CRAN.R-project.org/package=lme4]

