# Supplementary material for: Sexual dimorphism and natural variation within and among species in the Drosophila retinal mosaic
Source: BMC Evol Biol. 2014 Nov 26;14:240. doi: 10.1186/s12862-014-0240-x (PMC4268811; doi:10.1186/s12862-014-0240-x)
Supplement: Additional file 1: Table S1 — Counts of all ommatidia. [file 12862_2014_240_MOESM1_ESM.pdf]

Table S1. Counts of all ommatidia

species	strain	sex	Retina ID	total	DRA	pale	yellow	proportion p+OC(%)
D. mauritiana	TAM16	fem	9	880	42	342	496	40.8
			28	939	34	363	541	40.2
			29	902	41	377	484	43.8
		male	30	881	45	251	584	30.1
			32	897	38	233	605	27.8
			33	863	37	255	571	30.9
D. simulans	ZOM4	fem	11	866	36	327	502	39.4
			26	878	39	372	464	44.5
			27	925	46	430	448	49.0
		male	38	810	39	322	449	41.8
			39	803	38	317	442	41.8
			40	864	40	317	505	38.6
D. melanogaster	OreR	fem	22	752	41	269	442	37.8
			24	773	35	299	434	40.8
			25	767	45	263	459	36.4
		male	41	719	41	191	482	28.4
			42	698	35	201	457	30.5
			43	682	42	185	454	29.0
	Zi372	fem	44	861	39	331	484	40.6
			47	864	43	334	487	40.7
			48	892	40	339	502	40.3
		male	46	761	44	221	481	31.5
