# Supplementary figures and images for: Sexual dimorphism and natural variation within and among species in the Drosophila retinal mosaic
Source: BMC Evol Biol. 2014 Nov 26;14:240. doi: 10.1186/s12862-014-0240-x (PMC4268811; doi:10.1186/s12862-014-0240-x)

total number of ommatidia

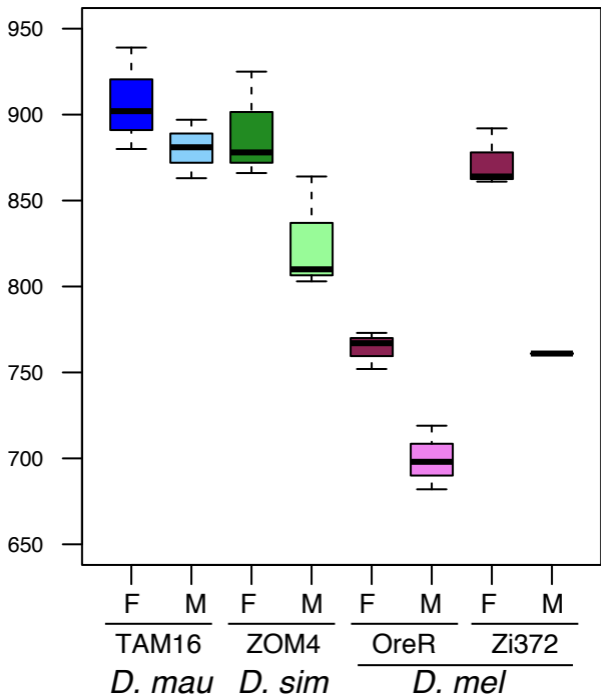

Supplement: Additional file 2: Figure S1 — Total ommatidium number per strain and sex. [file 12862_2014_240_MOESM2_ESM.pdf]

female

Retina ID: 09

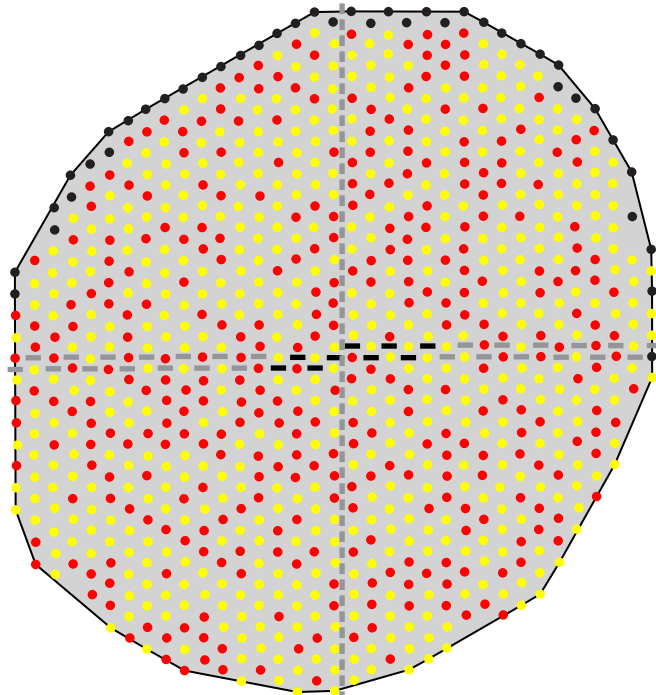

retina ID: 28

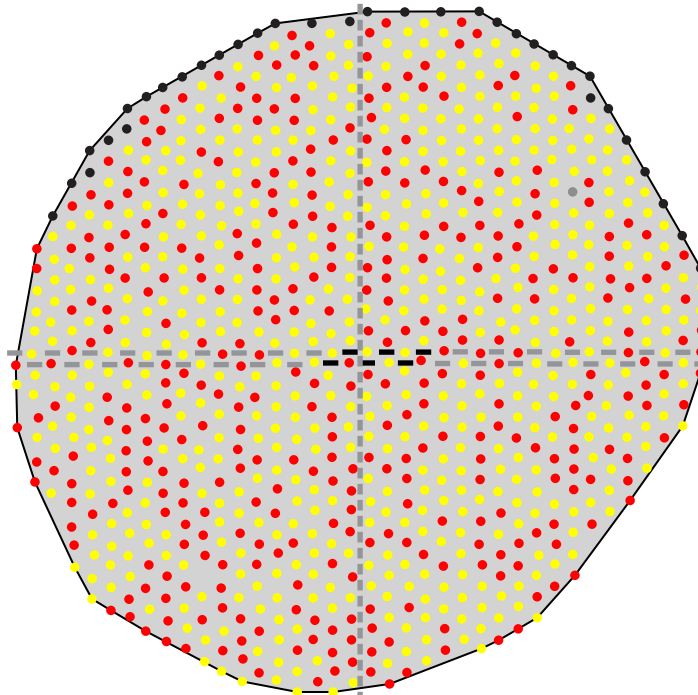

retina ID: 29

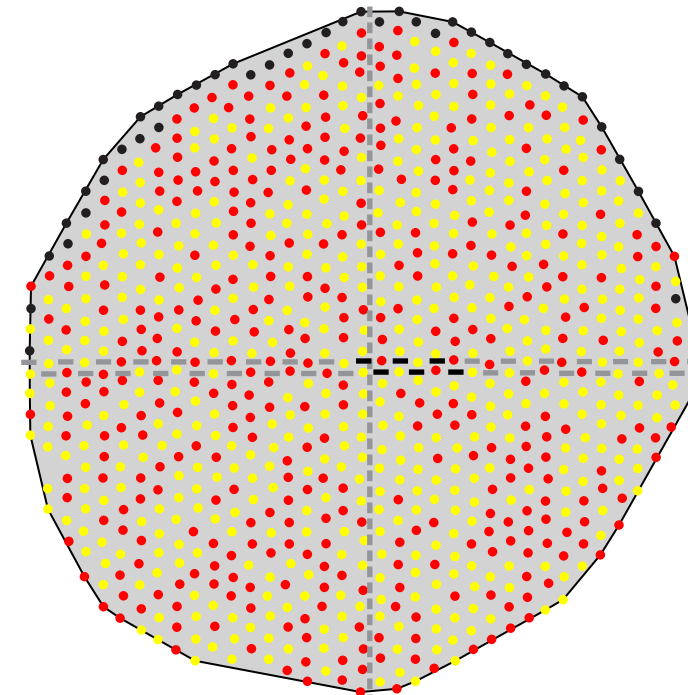

retina ID: 30

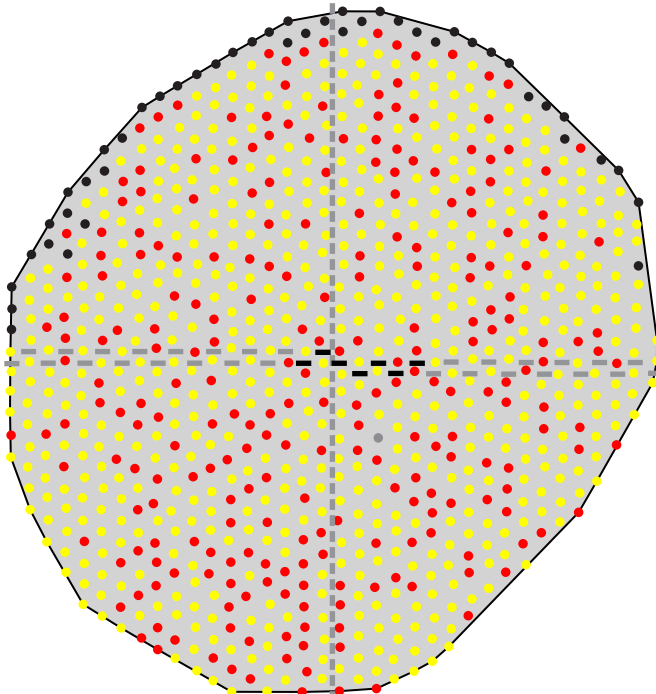

retina ID: 32

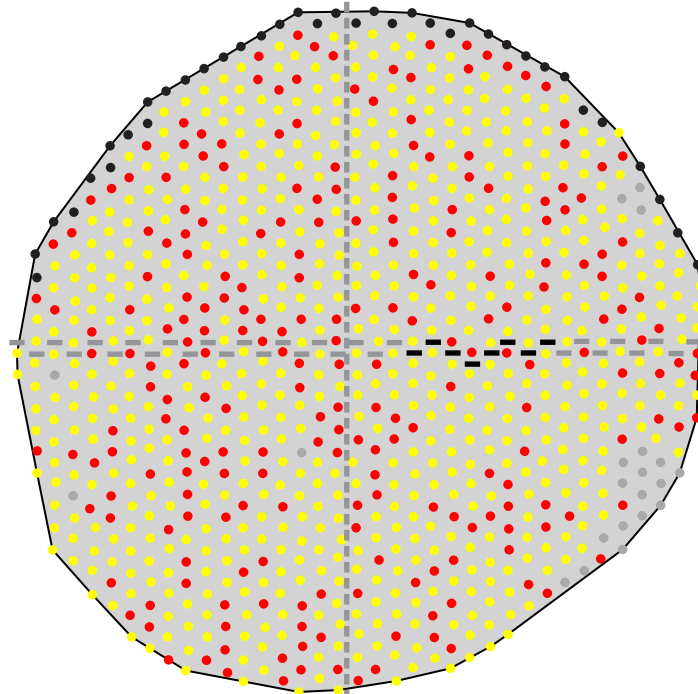

retina ID: 33

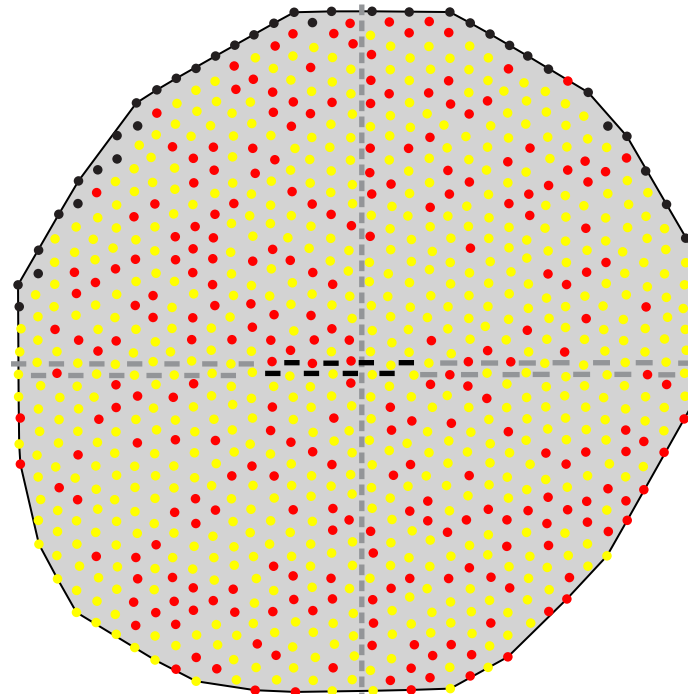

male

Supplement: Additional file 3: Figure S2 — Overview of D. mauritiana TAM16 retinal mosaic maps. [file 12862_2014_240_MOESM3_ESM.pdf]

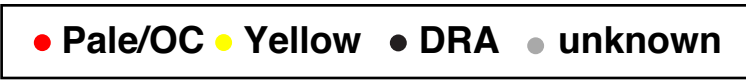

female

retina ID: 11

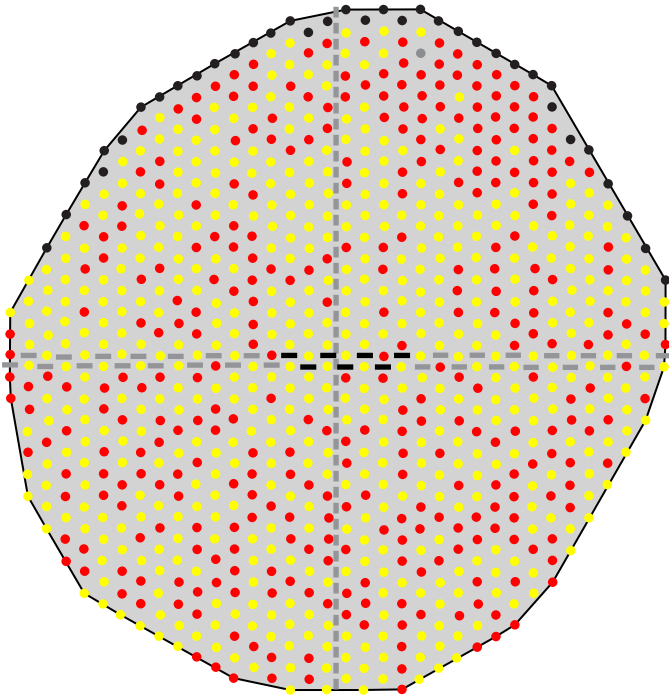

retina ID: 26

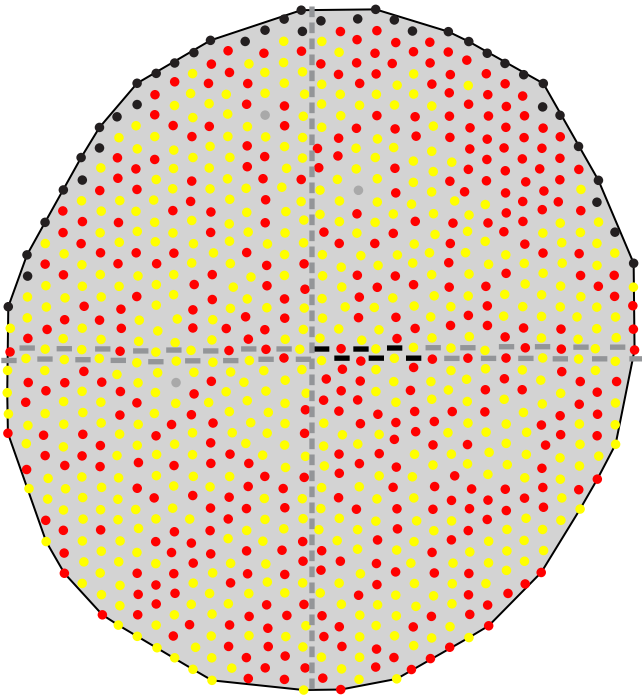

retina ID: 27

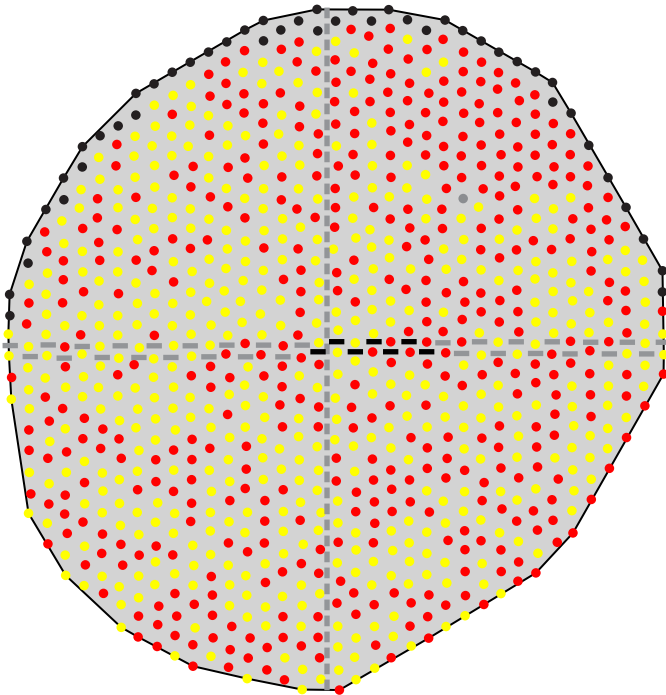

male

retina ID: 38

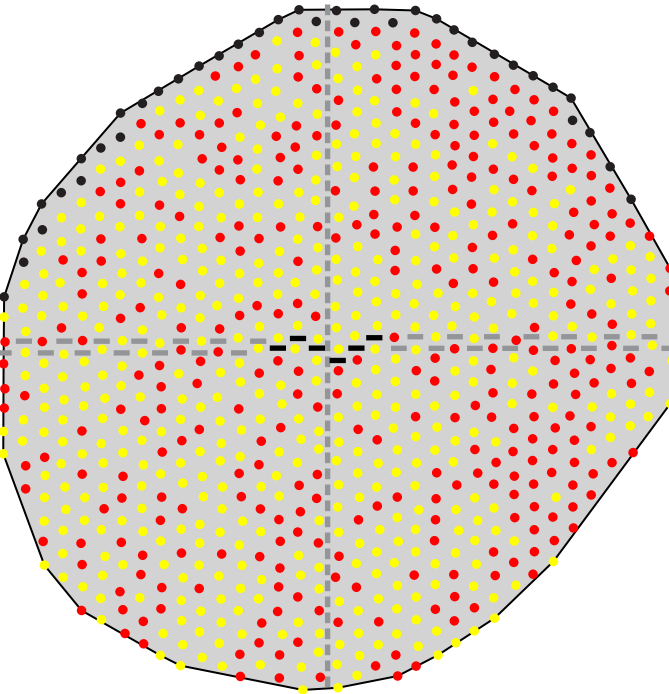

retina ID: 39

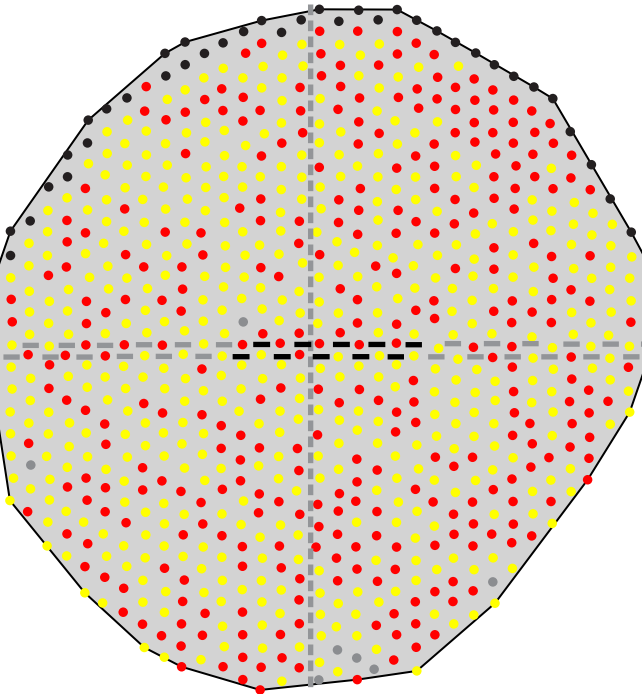

retina ID: 40

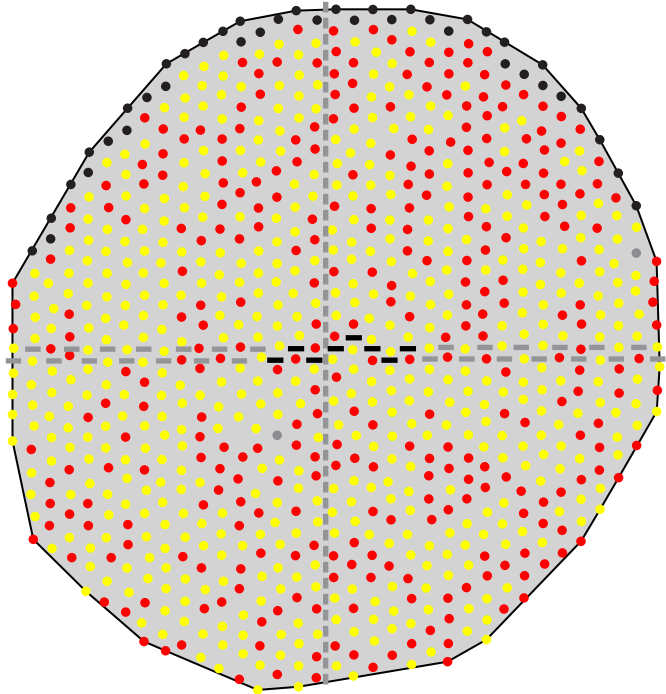

Supplement: Additional file 4: Figure S3 — Overview of D. simulans ZOM4 retinal mosaics. [file 12862_2014_240_MOESM4_ESM.pdf]

female

retina ID: 22

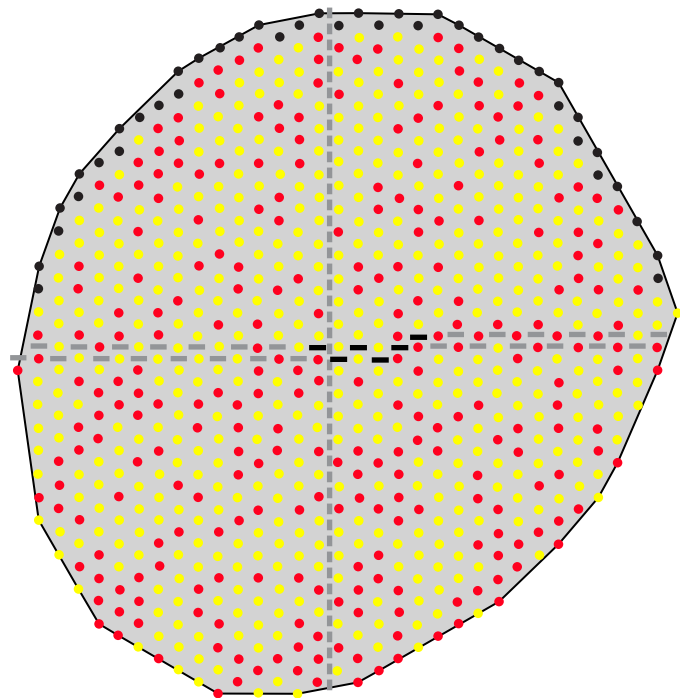

retina ID: 24

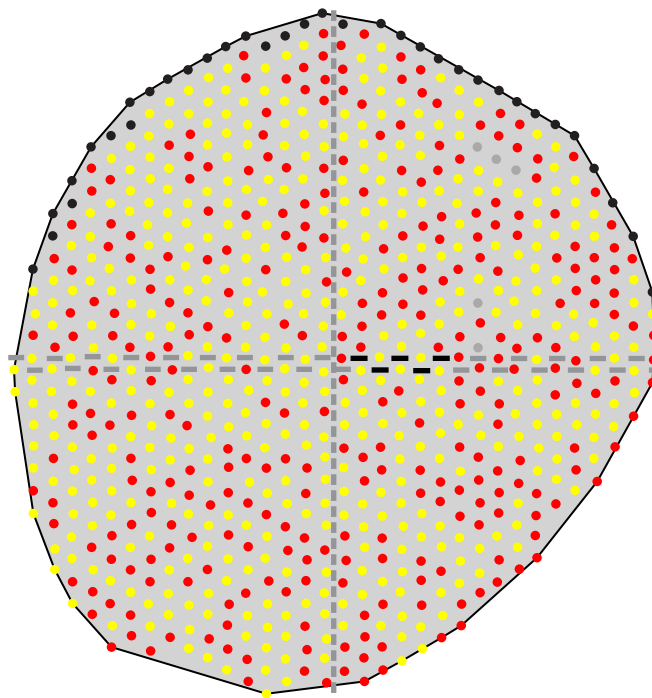

retina ID: 25

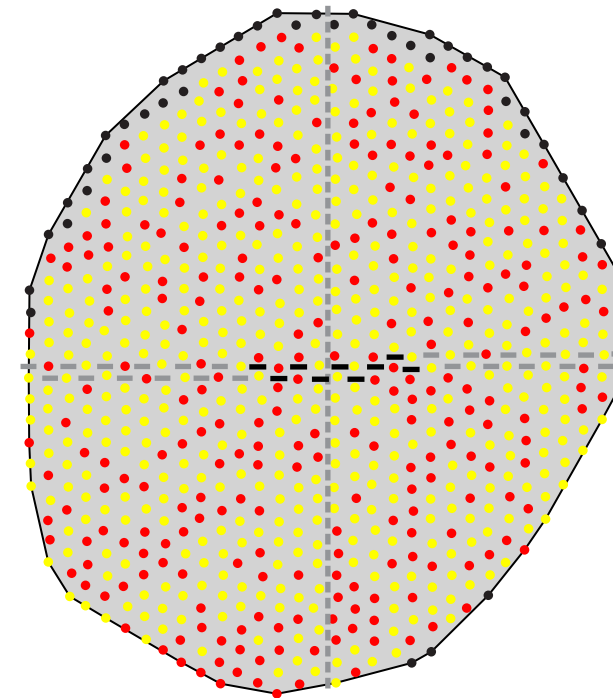

male

retina ID: 41

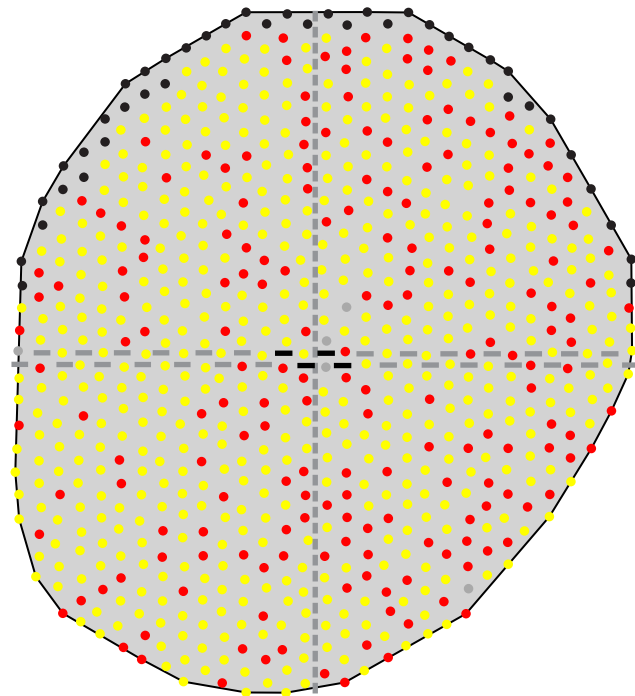

retina ID: 42

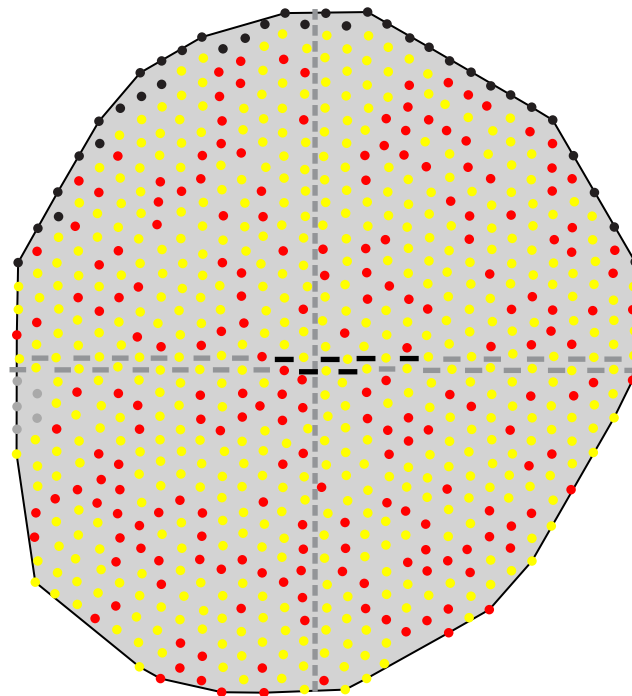

retina ID: 43

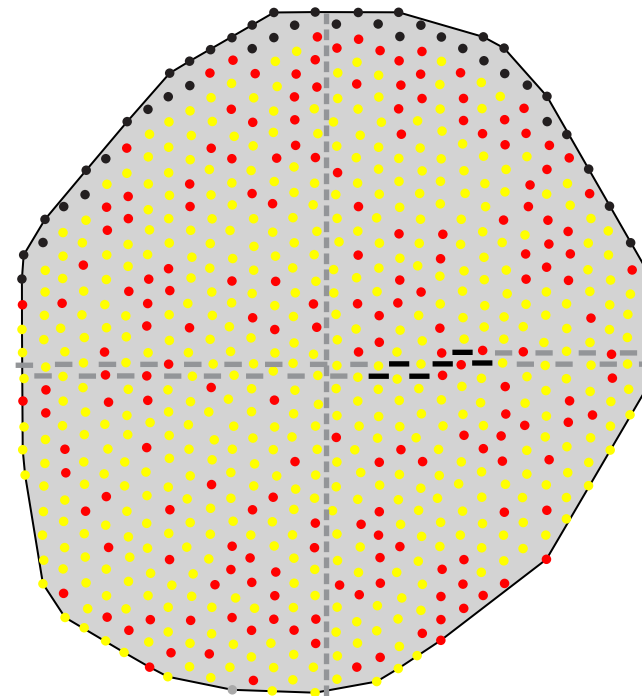

Supplement: Additional file 5: Figure S4 — Overview of D. melanogaster Oregon-R retinal mosaic maps. [file 12862_2014_240_MOESM5_ESM.pdf]

retina ID: 44

retina ID: 47

retina ID: 48

female

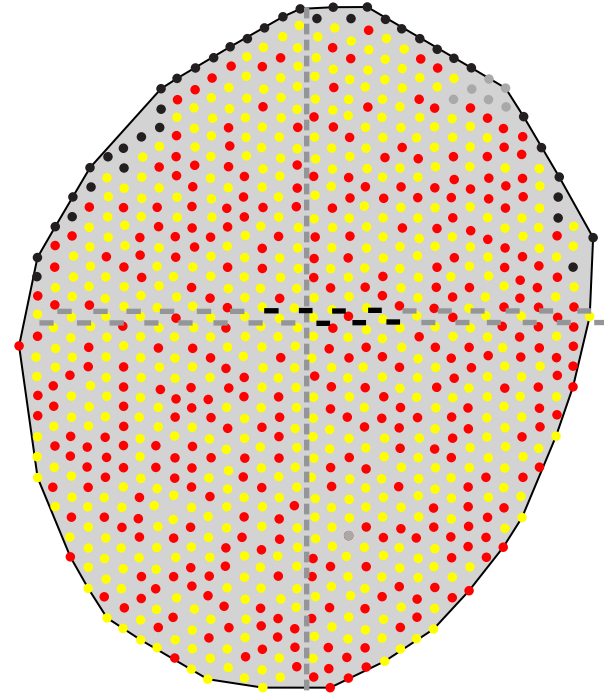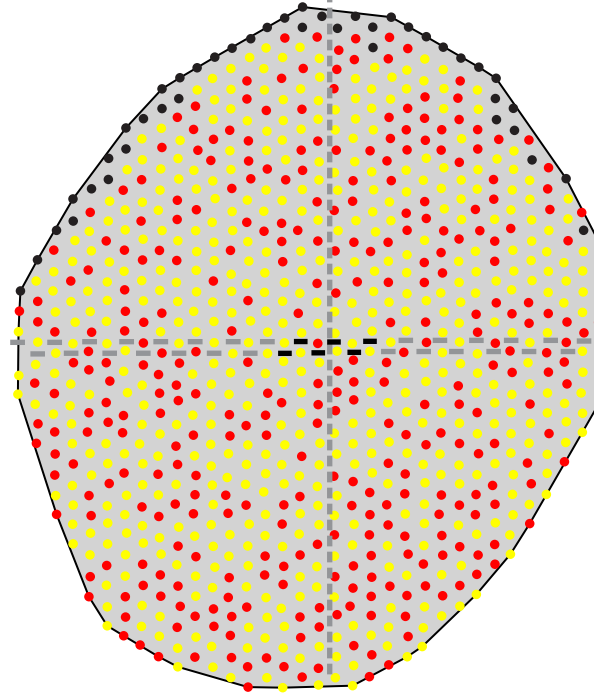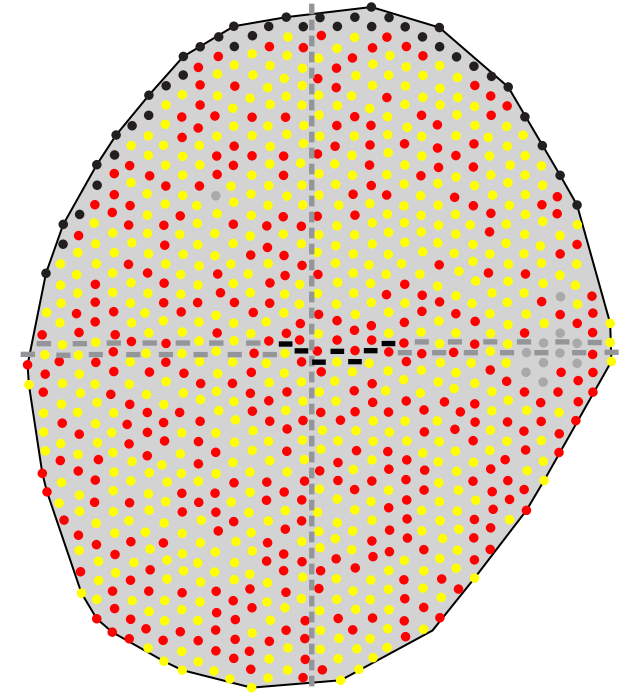

retina ID: 46

male

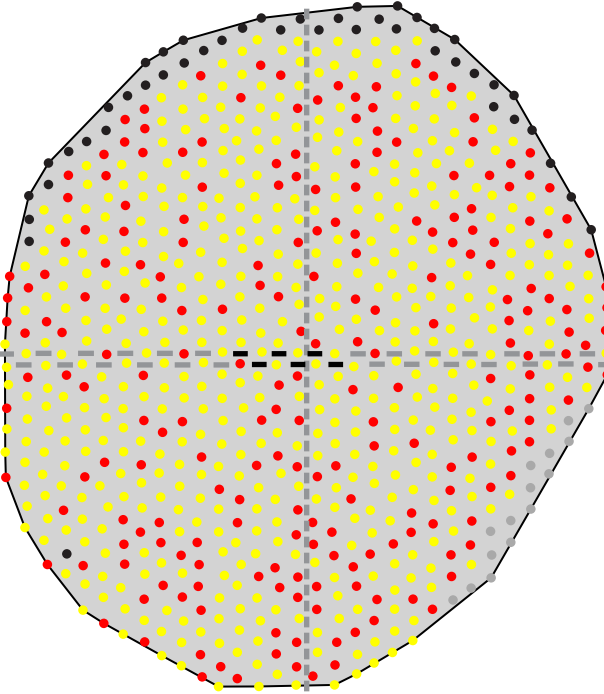

Supplement: Additional file 6: Figure S5 — Overview of D. melanogaster Zi372 retinal mosaic maps. [file 12862_2014_240_MOESM6_ESM.pdf]

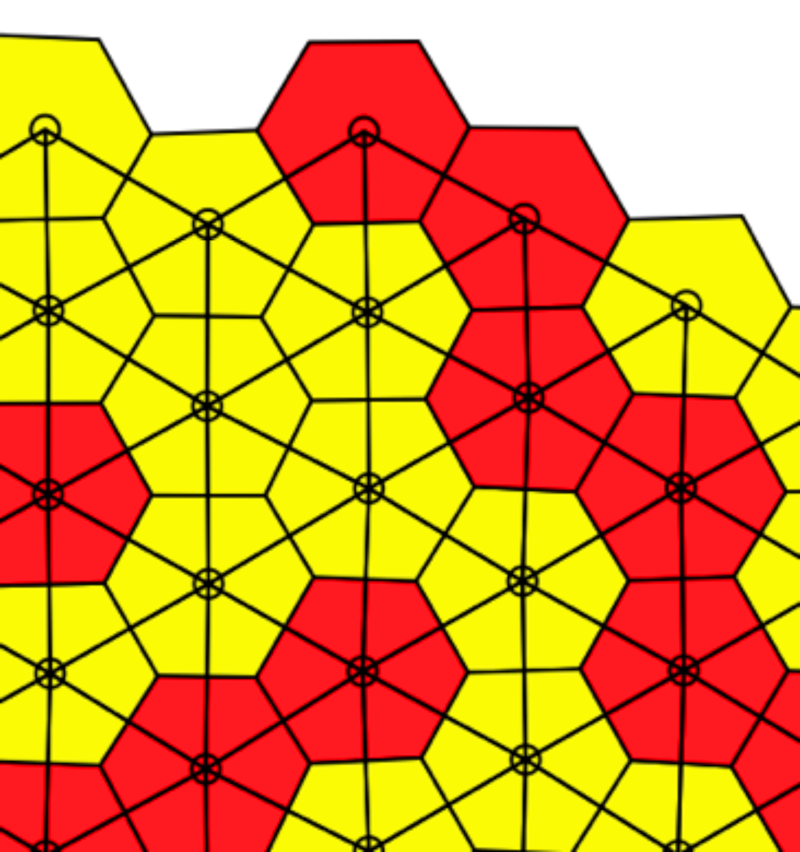

Supplement: Additional file 7: Figure S6 — Detail of a retinal mosaic map. Lines connecting closed circles indicate joins between contiguous ommatidia. [file 12862_2014_240_MOESM7_ESM.png]

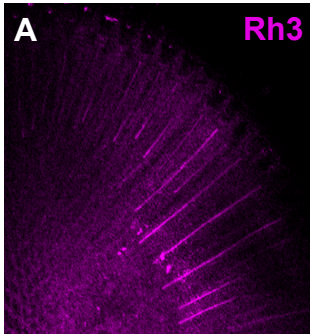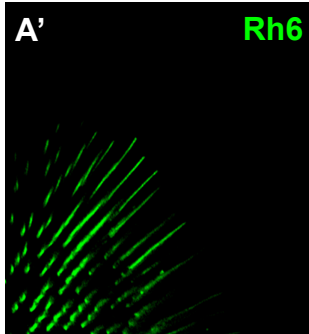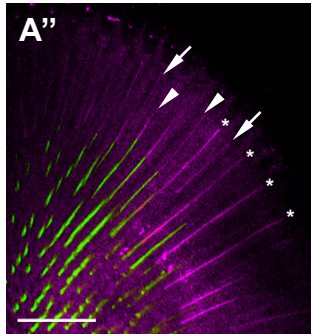

Supplement: Additional file 10: Figure S7 — Pale and Odd-Coupled ommatidia in the D. simulans ZOM4 dorsal-posterior retina. Combined immunostaining of Rh3 in R7 cells (left, magenta) and Rh6 in R8 cells (centre, green) show that the patch of p + OC ommatidia identified in the dorsal posterior retina of this strain consists of both p and OC ommatidia (right, overlay). Arrowheads: OC ommatidia, arrows: p ommatidia, asterisks: DRA ommatidia. Scale bar: 50 μm. [file 12862_2014_240_MOESM10_ESM.pdf]

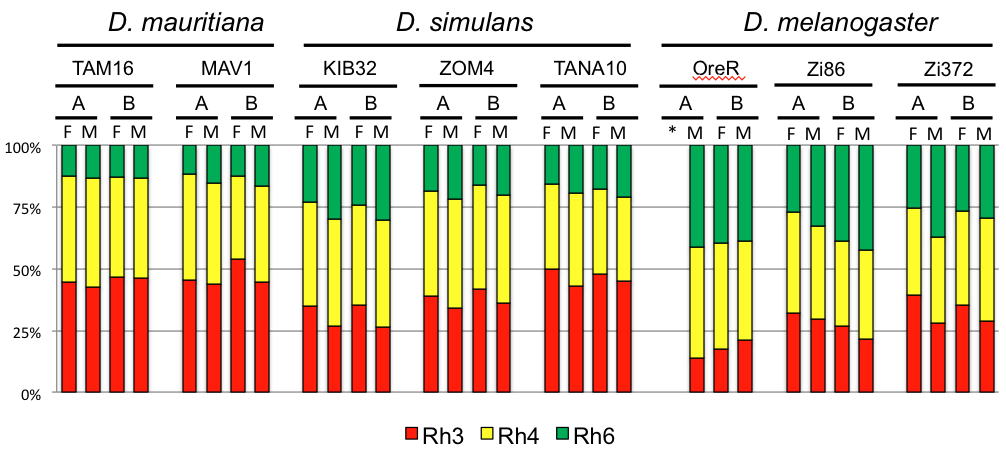

Supplement: Additional file 14: Figure S8 — Sexual dimorphism and variation between Drosophila species and strains in the expression of rh3, rh4 and rh6 mRNA. Histograms of the relative expression of rh3, rh4 and rh6, as a percentage of the total expression of these three rhodopsins, provide an alternative representation of the quantitative real-time PCR shown in Figure 5 of the main text. Compare with Figure 4 in Posnien et al. (2012), in which OreR represented D. melanogaster, Kib32 D. simulans and TAM16 D. mauritiana. [file 12862_2014_240_MOESM14_ESM.png]

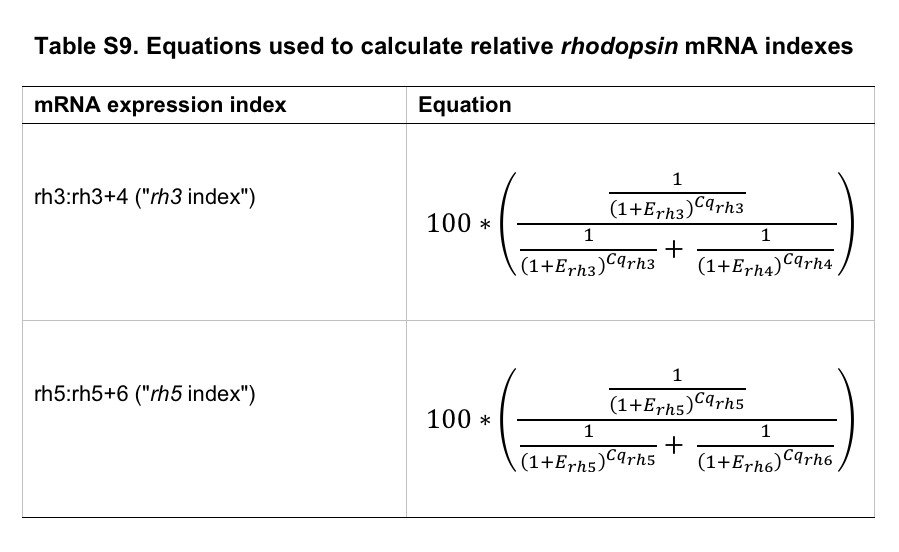

Supplement: Additional file 17: Table S9 — Equations used to calculate relative rhodopsin mRNA indexes. [file 12862_2014_240_MOESM17_ESM.png]
