# Supplementary material for: Sexual dimorphism and natural variation within and among species in the Drosophila retinal mosaic
Source: BMC Evol Biol. 2014 Nov 26;14:240. doi: 10.1186/s12862-014-0240-x (PMC4268811; doi:10.1186/s12862-014-0240-x)
Supplement: Additional file 8: Table S2 — Global spatial autocorrelation test statistics. [file 12862_2014_240_MOESM8_ESM.pdf]

Table S2. Global spatial autocorrelation test statistics

strain	sex	retina ID	join	Join count	Expected	Variance	z-value	p-value
TAM16	fem	9	p:p	396	398.5	157.1	-0.20	0.42
			y:y	838	843.9	176.9	-0.44	0.33
			y:p	1171	1162.7	561.6	0.35	0.37
		28	p:p	416	419.0	167.5	-0.23	0.41
			y:y	947	931.6	189.4	1.12	0.21
			y:p	1240	1252.4	602.9	-0.51	0.31
		29	p:p	472	473.6	170.8	-0.12	0.45
			y:y	804	781.1	184.9	1.69	0.10
			y:p	1198	1219.3	598.5	-0.87	0.19
	male	30	p:p	218	215.5	114.6	0.23	0.39
			y:y	1176	1169.5	156.4	0.52	0.35
			y:p	998	1007.0	437.2	-0.43	0.33
		32	p:p	194	184.1	103.7	0.97	0.25
			y:y	1260	1244.6	150.0	1.26	0.18
			y:p	935	960.3	403.4	-1.26	0.10
		33	p:p	234	225.4	116.6	0.80	0.29
			y:y	1155	1132.4	153.9	1.82	0.08
			y:p	982	1013.2	443.8	-1.48	0.07
ZOM4	fem	11	p:p	399	369.8	152.0	2.37	*0.02
			y:y	906	872.4	175.0	2.54	*0.02
			y:p	1076	1138.8	545.5	-2.69	**0.00
		26	p:p	493	473.1	167.3	1.54	0.12
			y:y	774	736.5	179.6	2.80	**0.01
			y:p	1126	1183.4	582.4	-2.38	**0.01
		27	p:p	648	602.8	184.3	3.33	**<0.0
			y:y	721	657.4	186.8	4.65	***<0.
			y:p	1153	1261.8	627.1	-4.35	***<0.
	male	38	p:p	418	384.8	147.5	2.73	**0.01
			y:y	811	752.2	163.9	4.59	***<0.
			y:p	987	1078.9	523.9	-4.02	***<0.
		39	p:p	397	378.4	147.0	1.53	0.12
			y:y	765	739.7	165.1	1.97	0.06
			y:p	1017	1060.9	515.5	-1.94	*0.03
		40	p:p	374	348.5	147.5	2.10	*0.04
			y:y	947	891.1	172.5	4.26	***<0.
			y:p	1036	1117.4	531.5	-3.53	***<0.
OreR	fem	22	p:p	293	292.1	126.4	0.08	0.40
			y:y	804	784.0	150.5	1.63	0.10
			y:p	939	959.9	454.5	-0.98	0.16
		24	p:p	355	350.3	139.0	0.40	0.37
			y:y	761	729.1	157.7	2.54	*0.02
			y:p	977	1013.6	490.2	-1.65	*0.05
		25	p:p	259	275.3	123.2	-1.47	0.07
			y:y	836	833.6	148.8	0.19	0.39
			y:p	975	961.0	449.3	0.66	0.32
	male	41	p:p	158	153.6	85.3	0.48	0.36
			y:y	1004	981.2	122.3	2.06	*0.05
			y:p	752	779.2	330.0	-1.50	0.07
		42	p:p	179	174.5	91.8	0.47	0.36
			y:y	918	904.8	125.0	1.18	0.20
			y:p	780	797.7	348.5	-0.95	0.17
		43	p:p	163	152.4	83.1	1.16	0.20
			y:y	947	920.7	115.9	2.45	*0.02
			y:p	715	752.0	320.4	-2.07	*0.02
Zi372	fem	44	p:p	372	389.6	155.2	-1.41	0.08
			y:y	807	818.9	176.4	-0.89	0.19
			y:p	1162	1132.5	547.7	1.26	0.18
		47	p:p	377	387.5	156.7	-0.84	0.20
			y:y	833	827.9	180.0	0.38	0.37
			y:p	1141	1135.6	548.9	0.23	0.39
		48	p:p	403	392.9	159.3	0.80	0.29
			y:y	892	855.7	183.0	2.69	*0.01
			y:p	1116	1162.5	561.0	-1.96	*0.02
	male	46	p:p	195	197.7	101.6	-0.27	0.39
			y:y	959	942.9	135.6	1.39	0.15
			y:p	853	866.4	383.2	-0.69	0.25
