# Supplementary material for: Sexual dimorphism and natural variation within and among species in the Drosophila retinal mosaic
Source: BMC Evol Biol. 2014 Nov 26;14:240. doi: 10.1186/s12862-014-0240-x (PMC4268811; doi:10.1186/s12862-014-0240-x)
Supplement: Additional file 9: Table S3 — Test statistics for comparison between dorsal/ventral and anterior/posterior retina compartments. [file 12862_2014_240_MOESM9_ESM.pdf]

Table S3. Test statistics for comparison between dorsal/ventral and anterior/posterior retina compartments

Species	Strain	Gender	N	p values[1]	
				dorsal/ ventral	ant/post
D. mauritiana	TAM16	f	3	0.235	0.018 *
		m	3	0.665	0.069
D. simulans	ZOM4	f	3	0.642	<0.001 ***
		m	3	0.524	<0.001 ***
D. melanogaster	OreR	f	3	0.139	0.249
		m	3	0.813	0.023 *
D. melanogaster	Zi372	f	3	0.015 *	0.440
		m	1	0.901[2]	0.192[2]

[1] replicated G test for pooled data except for: [2] G test for one retina only.
