# Supplementary material for: Sexual dimorphism and natural variation within and among species in the Drosophila retinal mosaic
Source: BMC Evol Biol. 2014 Nov 26;14:240. doi: 10.1186/s12862-014-0240-x (PMC4268811; doi:10.1186/s12862-014-0240-x)
Supplement: Additional file 12: Table S5 — rh3:rh3 + 4 qPCR results: statistics of LMM analyses. [file 12862_2014_240_MOESM12_ESM.pdf]

Table S5. *rh3*:*rh3+4* qPCR results: statistics of LMM analyses

**OreR excluded**				**OreR included**			
REML criterion at convergence: 570.2				REML criterion at convergence: 654.3			
Number of obs: 111, groups: strain, 7; biorep, 2				Number of obs: 123, groups: strain, 8; biorep, 2			
Random effects:				Random effects:			
Groups	SD			Groups	SD		
strain	5.68			strain	7.43		
biorep	0.35			biorep	0.72		
Residual	3.01			Residual	3.22		
Fixed effects:				Fixed effects:			
	Est.	SE	t-value		Est.	SE	t-value
(Intercept)	55.48	4.07	13.63	(Intercept)	55.26	5.32	10.39
melanogaster	-8.62	5.73	-1.50	melanogaster	-13.59	6.83	-1.99
simulans	-4.68	5.23	-0.90	simulans	-4.69	6.83	-0.69
gender: male	-4.55	0.57	-7.96	gender: male	-4.10	0.59	-7.00

Linear mixed model fit by REML ['lmerMod']

Formula: **Rh3Percentage ~ species + gender + (1 | strain) + (1 | biorep)**
