# Supplementary material for: Sexual dimorphism and natural variation within and among species in the Drosophila retinal mosaic
Source: BMC Evol Biol. 2014 Nov 26;14:240. doi: 10.1186/s12862-014-0240-x (PMC4268811; doi:10.1186/s12862-014-0240-x)
Supplement: Additional file 13: Table S6 — rh5:rh5 + 6 qPCR results: statistics of LMM analyses. [file 12862_2014_240_MOESM13_ESM.pdf]

Table S6. ***rh5*****:*****rh5+6*** **qPCR results: statistics of LMM analyses**

**OreR excluded**				**OreR included**			
REML criterion at convergence: 591.3				REML criterion at convergence: 685.4			
Number of obs: 112, groups: strain, 7; biorep, 2				Number of obs: 124, groups: strain, 8; biorep, 2			
Random effects:				Random effects:			
Groups	SD			Groups	SD		
strain	4.81			strain	7.95		
biorep	0.40			biorep	0.49		
Residual	3.27			Residual	3.61		
Fixed effects:				Fixed effects:			
	Est.	SE	t-value		Est.	SE	t-value
(Intercept)	67.21	3.47	19.36	(Intercept)	66.72	5.68	11.75
melanogaster	-20.36	4.87	-4.18	melanogaster	-26.49	7.31	-3.62
simulans	-10.98	4.45	-2.47	simulans	-10.95	7.31	-1.50
gender: male	-8.62	0.62	-13.95	gender: male	-7.64	0.65	-11.73

Linear mixed model fit by REML ['lmerMod']

Formula: **Rh5Percentage ~ species + (1 | strain) + (1 | biorep) + gender**
