# Supplementary material for: Sexual dimorphism and natural variation within and among species in the Drosophila retinal mosaic
Source: BMC Evol Biol. 2014 Nov 26;14:240. doi: 10.1186/s12862-014-0240-x (PMC4268811; doi:10.1186/s12862-014-0240-x)
Supplement: Additional file 15: Table S7 — Rhodopsin qPCR primer combinations. [file 12862_2014_240_MOESM15_ESM.pdf]

Table S7. Rhodopsin qPCR primer combinations

target	fw primer	rev primer	species	E
rh3	CCCGGAGCCACAATGATTC	CTTATGGCGTACACGAACGG	*mau,sim,mel*	0.89
rh4	TCCTGCCGCTAACCCAGTT	GAAGGAGCAGGACGTGAGG	*mau,sim,mel*	0.88
rh5	ACTCCGTTCGTGCTG	GGAGCAGGTGGTCAGGAAG	*mau,sim,mel*	0.86
rh6	CATTGTATACGGATTGAGCCA	CCAGGCACGGCATCTTCTC	*mau*	0.87
rh6	CATTGTGTACGGATTGAGCCA	CCAGGCACGGCATCTTCTC	*sim*	0.88
rh6	CATTGTGTACGGATTGAGCCA	CCAGGCATGGCATCTTCTC	*mel*	0.86

E: qPCR efficiency.
