# Supplementary material for: Sexual dimorphism and natural variation within and among species in the Drosophila retinal mosaic
Source: BMC Evol Biol. 2014 Nov 26;14:240. doi: 10.1186/s12862-014-0240-x (PMC4268811; doi:10.1186/s12862-014-0240-x)
Supplement: Additional file 16: Table S8 — qPCR thermo cycling program. [file 12862_2014_240_MOESM16_ESM.pdf]

Table S8. qPCR thermo cycling program

step	temp (deg C)	duration (min)	comments
1	50.0	2:00	UNG digest
2	95.0	10:00	activation
3	95.0	0:10	denaturation
4	57.0	0:30	annealing
5 (->goto 3, 39x)	72.0	0:15	elongation+ plate read
